# Water-soluble saponins accumulate in drought-stressed switchgrass and may inhibit yeast growth during bioethanol production

**DOI:** 10.1186/s13068-022-02213-y

**Published:** 2022-10-31

**Authors:** Sarvada Chipkar, Katherine Smith, Elizabeth M. Whelan, Derek J. Debrauske, Annie Jen, Katherine A. Overmyer, Andrea Senyk, Larkin Hooker-Moericke, Marissa Gallmeyer, Joshua J. Coon, A. Daniel Jones, Trey K. Sato, Rebecca G. Ong

**Affiliations:** 1 Department of Chemical Engineering, Michigan Technological University, 1400 Townsend Drive, Houghton, MI USA; 2 DOE Great Lakes Bioenergy Research Center, University of Wisconsin-Madison, Madison, WI USA; 3 DOE Great Lakes Bioenergy Research Center, Michigan Technological University, Houghton, MI USA; 4 RTSF Mass Spectrometry & Metabolomics Core, Michigan State University, East Lansing, MI USA; 5 Department of Biochemistry and Molecular Biology, Michigan State University, East Lansing, MI USA; 6 Department of Biomolecular Chemistry, University of Wisconsin-Madison, Madison, WI USA; 7 Department of Chemistry, University of Wisconsin-Madison, Madison, WI USA; 8 Morgridge Institute for Research, Madison, WI USA

**Keywords:** Drought, Fermentation inhibition, Water-extraction, Saponins, Switchgrass

## Abstract

**Background:**

Developing economically viable pathways to produce renewable energy has become an important research theme in recent years. Lignocellulosic biomass is a promising feedstock that can be converted into second-generation biofuels and bioproducts. Global warming has adversely affected climate change causing many environmental changes that have impacted earth surface temperature and rainfall patterns. Recent research has shown that environmental growth conditions altered the composition of drought-stressed switchgrass and directly influenced the extent of biomass conversion to fuels by completely inhibiting yeast growth during fermentation. Our goal in this project was to find a way to overcome the microbial inhibition and characterize specific compounds that led to this inhibition. Additionally, we also determined if these microbial inhibitors were plant-generated compounds, by-products of the pretreatment process, or a combination of both.

**Results:**

Switchgrass harvested in drought (2012) and non-drought (2010) years were pretreated using Ammonia Fiber Expansion (AFEX). Untreated and AFEX processed samples were then extracted using solvents (i.e., water, ethanol, and ethyl acetate) to selectively remove potential inhibitory compounds and determine whether pretreatment affects the inhibition. High solids loading enzymatic hydrolysis was performed on all samples, followed by fermentation using engineered *Saccharomyces cerevisiae*. Fermentation rate, cell growth, sugar consumption, and ethanol production were used to evaluate fermentation performance. We found that water extraction of drought-year switchgrass before AFEX pretreatment reduced the inhibition of yeast fermentation. The extracts were analyzed using liquid chromatography–mass spectrometry (LC–MS) to detect compounds enriched in the extracted fractions. Saponins, a class of plant-generated triterpene or steroidal glycosides, were found to be significantly more abundant in the water extracts from drought-year (inhibitory) switchgrass. The inhibitory nature of the saponins in switchgrass hydrolysate was validated by spiking commercially available saponin standard (protodioscin) in non-inhibitory switchgrass hydrolysate harvested in normal year.

**Conclusions:**

Adding a water extraction step prior to AFEX-pretreatment of drought-stressed switchgrass effectively overcame inhibition of yeast growth during bioethanol production. Saponins appear to be generated by the plant as a response to drought as they were significantly more abundant in the drought-stressed switchgrass water extracts and may contribute toward yeast inhibition in drought-stressed switchgrass hydrolysates.

**Supplementary Information:**

The online version contains supplementary material available at 10.1186/s13068-022-02213-y.

## Background

There is an ever-increasing demand for non-conventional energy resources with the fast depletion of natural petroleum reservoirs. The global scientific community has been on a mission to discover alternate ways to harness energy from different sources, including lignocellulosic biomass [1–4]. Switchgrass, a perennial prairie grass native to the United States, has been identified as a potential bioenergy feedstock [5, 6]. When grown on non-arable land, using switchgrass for bioenergy production mitigates the food vs. fuel debate usually observed with food-based feedstocks, such as corn and sugarcane [3, 4]. However, several challenges exist toward achieving a viable lignocellulosic biofuel industry, one of which is variability in feedstock composition. Due to rapid global warming, changing weather conditions have led to the frequent occurrence of abiotic stresses, such as drought, wildfire, water salinity, and biotic stresses, such as pathogen infections or herbivore attack. These stresses reduce the amount of available biomass and alter the feedstock composition [6–8].

Many biotic and abiotic stresses trigger the generation of plant defense compounds that ensure higher plant growth and survival. Beans, olives, wild geophytes, and grasses have been found to generate compounds, such as flavonoids, alkaloids, terpenes, phenols, anthocyanins, tannins, and quinones in response to drought [9–12]. Osmolytes in the form of amino acids including proline, glycine, and betaine are also commonly observed in plants surviving a drought [13, 14]. Specifically, switchgrass varieties that endured drought conditions possessed higher fructose, trehalose, abscisic acid, and spermine [6, 15, 16]. In our prior work, we showed that the effect of switchgrass harvested during U.S. Midwestern drought of 2012 carried through the biofuel production process to affect fermentation [17]. In that study, hydrolysates generated from switchgrass grown during the Midwestern U.S. drought of 2012 completely inhibited the growth of *Saccharomyces cerevisiae* [17].

Microbial inhibitors obtained from biomass can be broadly classified into four categories: carbohydrate degradation products, lignin degradation products, pretreatment chemicals and derivatives, and naturally generated plant-defense compounds. Carbohydrate degradation inhibitors including furfural and hydroxymethylfurfural (HMF) are commonly generated during dilute acid, hot water, or steam explosion pretreatments [18]. Imidazoles and pyrazines are formed from soluble sugars via Maillard reactions during ammonia fiber expansion (AFEX) pretreatment [19] and may contribute toward yeast fermentation inhibition [20]. Lignin-derived inhibitors are typically phenolic molecules generated from alkali and strongly acidic pretreatment methods [18, 21–23]. Pretreatment extraction methods, such as gamma-valerolactone and ionic liquids may leave residual toxic chemicals that adversely affect the fermentation microbes [24, 25]. Plant-defense compounds may be formed in response to ambient biotic and abiotic stresses, including osmolytes, phenolic glycosides, flavonoids, tannins, and alkaloids, and constitute a fourth category of microbial inhibitors [11, 12]. Phenolic metabolites including rutin, quercetin, gallic acid, hydroxycinnamic acid, and triterpene glycosides, or saponins, have been found in switchgrass, and are known to possess antioxidant and antibacterial properties [16, 26–29]. If these compounds survive pretreatment and enzymatic hydrolysis, they could feasibly inhibit microbial fermentation.

The objective of this paper was to determine which classes of compounds are responsible for inhibition of yeast cultured in drought-stressed switchgrass hydrolysates. Switchgrass from drought and non-drought years were pretreated using ammonia fiber expansion (AFEX), and untreated and AFEX-treated switchgrass were extracted separately with three different solvents to selectively remove potential inhibitory compounds [30, 31]. To determine which extractions alleviated the fermentation inhibition, high solids loading enzymatic hydrolysis was performed on all samples, followed by fermentation using genetically modified *Saccharomyces cerevisiae*. Liquid chromatography–mass spectrometry (LC–MS) was performed on the solvent extracts to identify compounds that were comparatively more abundant in the extracts whose removal alleviated the inhibition. Fermentation experiments were then conducted where functionally and chemically similar compounds were added to the control switchgrass hydrolysates to confirm the ability of the compounds to inhibit yeast growth.

## Results

### Solvent extraction increased sugar yields from AFEX-treated switchgrass

**Fig. 1:**
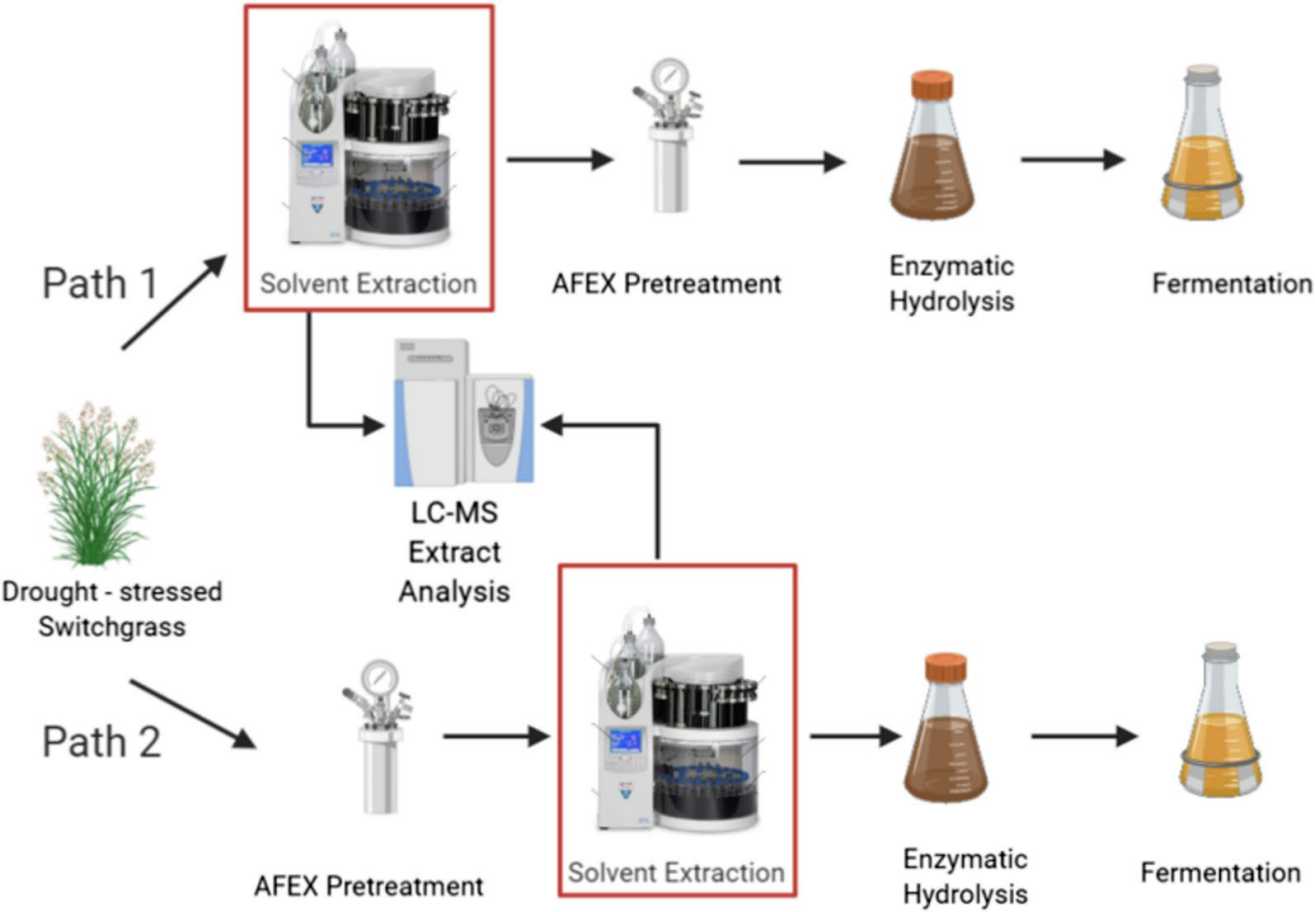
Yeast inhibitors produced in drought-stressed switchgrass were extracted using different solvents before and after pretreatment to trace the source of inhibitor generation, i.e., if the inhibitor(s) is a plant-defense compound or a by product of the pretreatment method. The flowchart was created using Biorender

Switchgrass harvested at the Arlington agricultural research station in 2010 (average rainfall year) and 2012 (drought year) were used in seven sets of hydrolysis experiments. To determine classes of compounds that might be responsible for yeast inhibition, extractions were separately performed on untreated and AFEX-treated switchgrass in triplicates for each solvent (water, ethanol, and ethyl acetate). The two extraction timings (before and after pretreatment) were employed to identify the source of the microbial inhibitor(s): Path 1—generated by the plant or Path 2—modified/produced by the pretreatment (Fig. 1). In brief, AFEX-pretreated biomass was enzymatically hydrolyzed, and the liquid was separated from the solids, which were discarded. The hydrolysates were then fermented using engineered yeast. LC–MS was used to characterise the inhibitors present in the extracted solvents. An unextracted control set was additionally processed to replicate the microbial inhibition reported in previous research [17] and create a baseline for the hydrolysis and fermentation experiments performed on extracted switchgrass.

Greater mass loss was observed for samples when they were extracted following pretreatment, compared to the untreated samples (Table 1), similar to previous observations by Ong et al. [32]. For the untreated samples, water and ethyl acetate extractions removed more mass from the control-year switchgrass, while ethanol extractions removed more mass from the drought-year switchgrass. There was more mass extracted from drought-year pretreated samples when compared to respective control samples for all other sample sets. The composition analysis of untreated and extracted biomass are reported in the supplementary document of the manuscript (Additional file 1: Tables S1, S2).

**Table 1:** Greater mass was extracted from switchgrass by all solvents after AFEX-pretreatment

Average mass extracted with respect to initial dry biomass loaded (g/100 g dry biomass)								
Extraction type	Untreated				AFEX-treated			
	2010 (Control)		2012 (Drought)		2010 (Control)		2012 (Drought)	
	Avg	SD	Avg	SD	Avg	SD	Avg	SD
Water	13.7	0.2	9.1	1.3	26.2	2.4	34.4	2.7
Ethanol	1.0	0.3	8.0	0.6	12.4	0.3	24.8	0.3
Ethyl acetate	7.1	0.7	15.4	1.1	13.5	0.3	22.3	0.9

‘Avg’ stands for average and ‘SD’ for standard deviation for *n* = 3

**Fig. 2:**
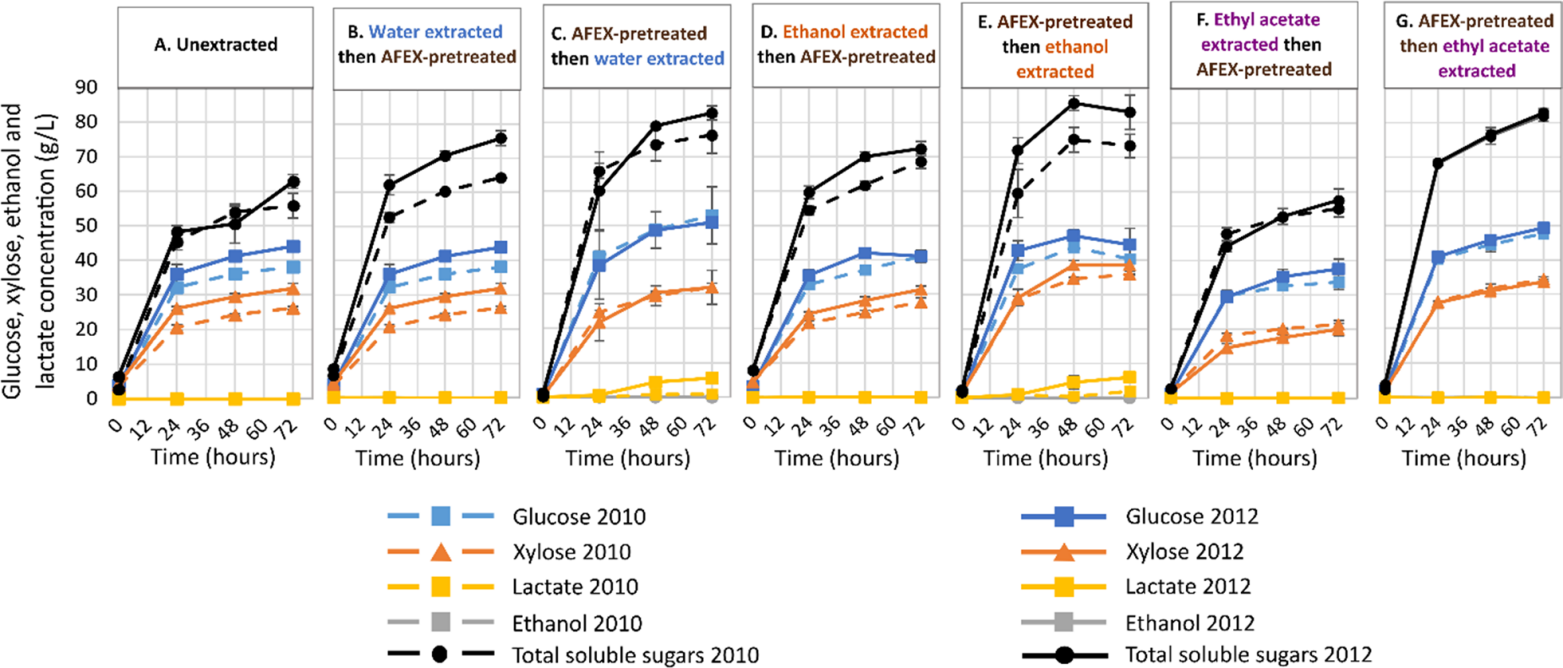
Production of glucose and xylose was significantly higher in switchgrass extracted after pretreatment (**C**, **E**, **G**) than the samples extracted before the pretreatment (**B**, **D**, **F**) for a specific solvent or the unextracted switchgrass (**A**), indicating removal of enzyme inhibitors for biomass extracted after AFEX pretreatment. Total soluble sugars are the sum of glucose and xylose. Data points represent the average ± standard deviation (*n* = 3)

**Fig. 3:**
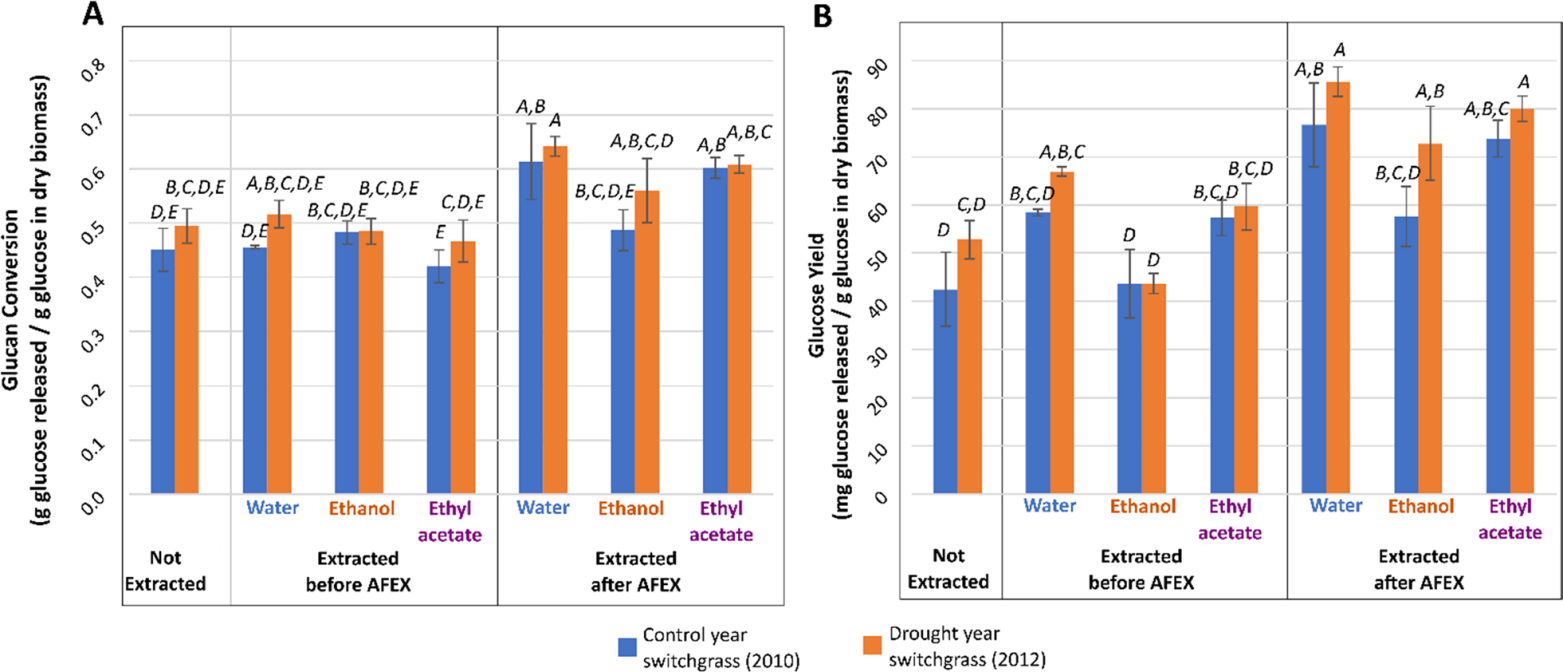
Glucan conversion (**A**) and glucose yield (**B**) for enzymatically hydrolyzed switchgrass after 72 h. Samples that do not share a letter on top of the bars are significantly different based on Tukey’s pairwise statistical comparison (⍺ = 0.05). In this statistical model, year and extraction solvent were nested within extraction timing with respect to AFEX pretreatment. Data points represent the average ± standard deviation (*n* = 3)

During high solids enzymatic hydrolysis (7% glucan loading), structural sugars such as glucan and xylan found in the plant cell wall were deconstructed into the fermentable sugars: glucose and xylose (Fig. 2). The reaction conditions for this experiment were previously optimized by Ong et al. and Chandrasekar et al. [17, 33]. There was slightly more glucose and xylose produced from switchgrass that had been extracted after AFEX pretreatment compared to the switchgrass that was extracted before pretreatment, for all solvents (Fig. 2B–G). This suggested that the extractions after AFEX pretreatment removed inhibitors of enzymatic hydrolysis, as indicated by the greater mass removal, resulting in a higher conversion rate of structural sugars into monomeric sugars [34–36]. Glucan conversion and glucose yield followed a similar trend for each set of solvent extracted samples i.e., the yield was higher for samples extracted with a specific solvent after pretreatment than that were extracted before the pretreatment (Fig. 3A, B; Additional file 1: Table S3A, B).

The antibiotic geneticin was used to control microbial contamination during enzymatic hydrolysis, though the presence of lactic acid indicates slight microbial contamination in the hydrolysates (Fig. 2C,E) [37]. No residual ethanol or ethyl acetate was detected in the ethanol- or ethyl acetate-extracted samples (Fig. 2D-G), which could have been inhibitory toward yeast during fermentation.

### Water extraction of untreated switchgrass was most effective in overcoming yeast inhibition

Fermentation experiments were performed on the switchgrass hydrolysates in a respirometer apparatus to monitor the carbon dioxide generated during fermentation. Engineered *Saccharomyces cerevisiae* GLBRCY945 was used for fermentation. Carbon dioxide can be used as an indicator of ethanol production in fermentation processes [38, 39] as consumption of glucose is related using the following stoichiometry:

$${\mathrm{C}}_{6}{\mathrm{H}}_{12}{\mathrm{O}}_{6} \to 2{\mathrm{C}}_{2}{\mathrm{H}}_{5}\mathrm{OH} +2{\mathrm{CO}}_{2}+2\mathrm{ATP}$$

This method has previously been used to successfully differentiate between the drought-year (2012) and non-drought year (2010) switchgrass hydrolysates as they have vastly different fermentation profiles [33].

**Fig. 4:**
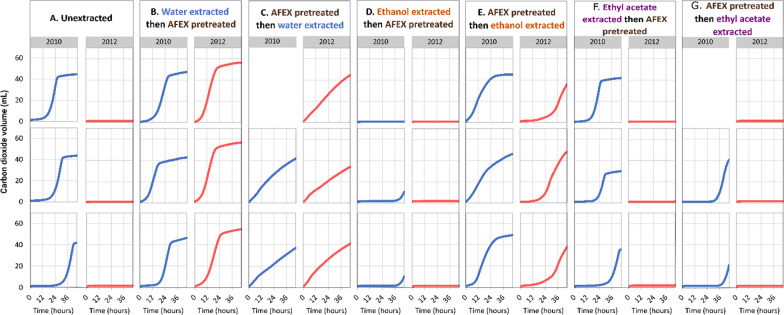
Carbon dioxide produced during yeast fermentation showed reproducible patterns across the different extraction treatments. **A** Unextracted switchgrass. **B** Pre-AFEX water extraction. **C** Post-AFEX water extraction. **D** Pre-AFEX ethanol extraction. **E** Post-AFEX ethanol extraction. **F** Pre-AFEX ethyl acetate extraction. **G** Post-AFEX ethyl acetate extraction. Rows represent replicates for a particular sample in a column, 2010 (normal year) and 2012 (drought year) in the same extraction column and same row were paired for all hydrolysis and fermentation experiments

Unextracted drought-stressed switchgrass (2012) produced no carbon dioxide over 45 h of fermentation, indicating complete yeast inhibition in the hydrolysate (Fig. 4A). This fermentation profile was in stark contrast to the switchgrass grown in the normal rainfall year (2010) based on the volume of carbon dioxide produced. These results align with previously reported results [17, 33] and provide a baseline for comparing the effects of extraction on yeast fermentation. Water extraction prior to AFEX completely alleviated yeast inhibition in the drought-year (2012) switchgrass, while the non-stress year (2010) showed comparable CO_2_ production as the unextracted samples and a shorter lag phase, indicating no nutrient limitation following water extraction (Fig. 4B). Lag phase is the period when the yeast adapts to its growth media before beginning to multiply exponentially. In contrast, switchgrass samples that were water extracted after AFEX-pretreatment had a reduction in the lag phase, with growth beginning immediately, but at a much slower rate compared to unextracted control-year samples (Fig. 4C). This peculiarity of the growth curve has been previously reported for *E. coli* and attributed to dilution of an inhibitory hydrolysate [40]. Removal of inhibitors in the pretreated biomass would correspond to an apparent dilution of inhibitors in the hydrolysate when loaded based on the glucan content, which was fixed at 7% in our experiments.

Control-year samples (2010) that were extracted with ethyl acetate before AFEX showed a similar CO_2_ production pattern as the unextracted samples, while the samples extracted with ethyl acetate after AFEX-treatment had a significant delay in carbon dioxide production (Fig. 4A, F, G)**.** Drought-year ethyl-acetate extracted switchgrass (Fig. 4F, G) showed total inhibition similar to unextracted drought-year switchgrass (Fig. 4A). There was no evidence of residual ethyl acetate in any of the switchgrass hydrolysates based on HPLC analysis, indicating that residual extraction solvent was not responsible for the observed result.

Ethanol extraction of pretreated switchgrass resulted in greater carbon dioxide production compared to the control (Fig. 4E). However, the lag phase for the yeast growth was longer in drought-year samples compared to the paired control-year samples. Switchgrass extracted with ethanol before AFEX-pretreatment showed no carbon dioxide generation for the drought year and a significant delay in carbon dioxide production for the control-year samples (Fig. 4D). There was no evidence of residual ethanol in any of the hydrolysates prior to fermentation (Fig. 3D, E), which could have inhibited the fermentation. It is possible the inhibition could be attributed to the removal of essential nutrients beneficial to the survival of *S. cerevisiae,* such as amino acids and other nitrogenous compounds. To investigate this possibility, 18 different amino acids were quantified for unextracted and water- and ethanol-extracted hydrolysates (Additional file 1: Fig. S1; Table S4A, B).

**Fig. 5:**
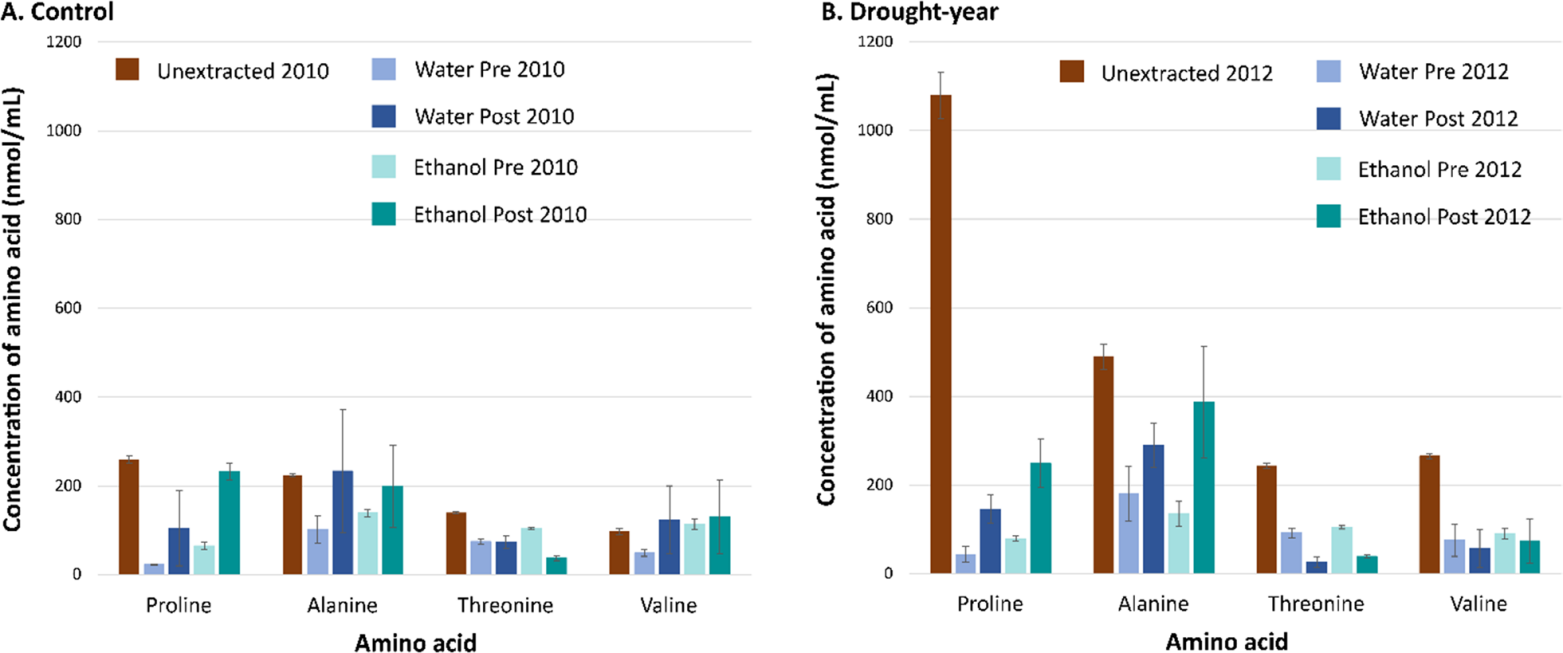
Within the same year—**A** control year (2010) or **B** drought year (2012)—proline, valine, alanine, and threonine contents were similar for ethanol and water extracted switchgrass hydrolysates. These amino acids were present in greater amounts in **B** unextracted drought-year switchgrass hydrolysates. The other amino acids showed similar trends. ‘Pre’ stands for samples that were extracted then AFEX-pretreated while ‘Post’ for samples that were AFEX-pretreated then extracted

As expected, osmolytes such as proline, alanine, valine and, threonine [6, 13, 15] were seen in higher amounts in unextracted drought-year samples compared to control samples (Fig. 5). The 18 different amino acid concentrations were statistically similar for water-extracted (non-inhibitory) and ethanol extracted (inhibitory) untreated switchgrass hydrolysates (Additional file 1: Table S4A, B). However, these were statistically lower when compared to unextracted samples. This indicates that the yeast inhibition observed in ethanol extracted hydrolysates (Fig. 4C) was not due to limitations in hydrolysate amino acid content.

**Fig. 6:**
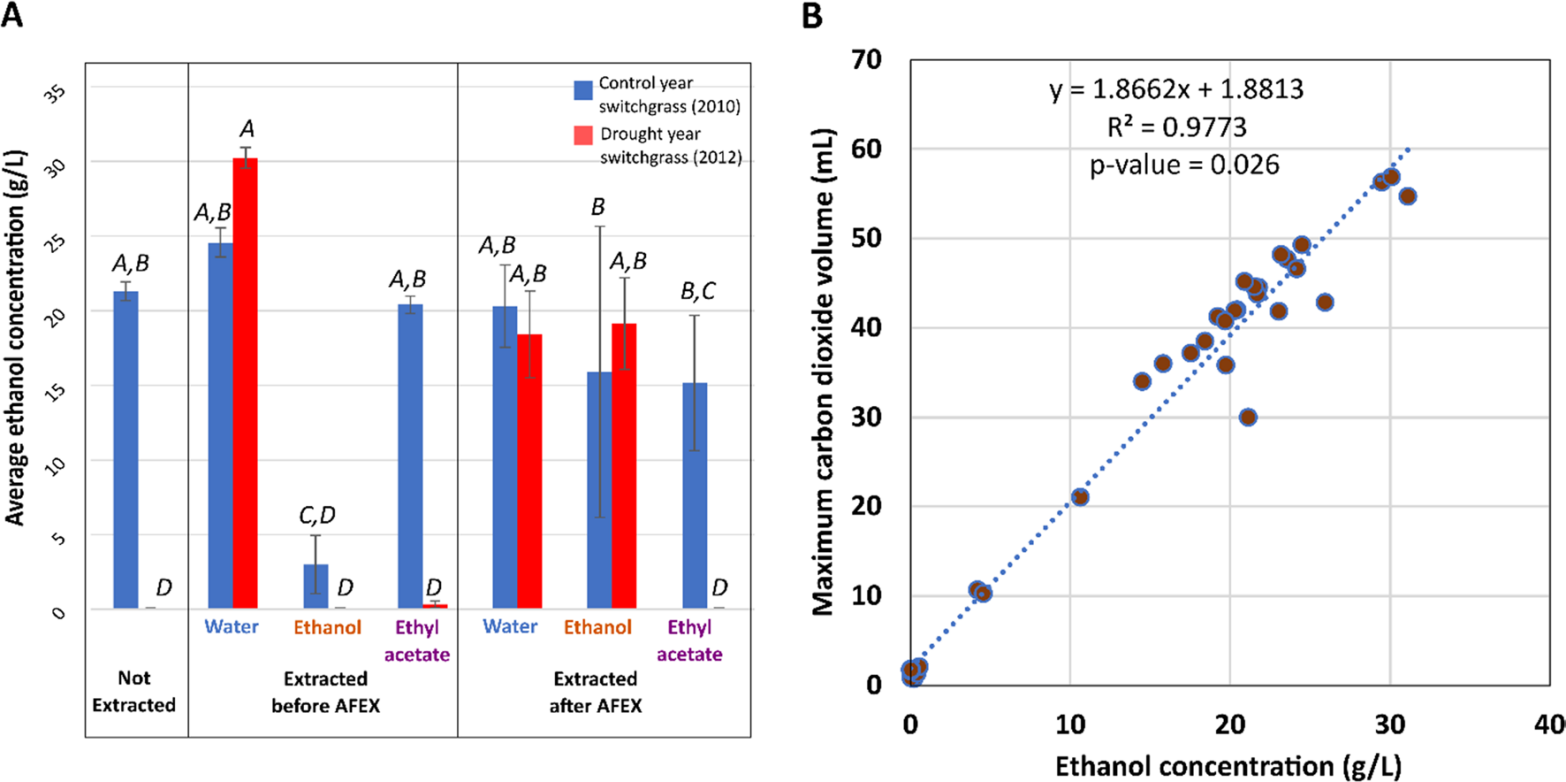
Final ethanol concentrations strongly correlate with maximum carbon dioxide volume across all samples. **A** Average ethanol concentration at the end of respirometer experiments for extracted and unextracted switchgrass hydrolysates. Samples that do not share a letter on top of the bars are significantly different based on Tukey’s pairwise statistical comparison (⍺ = 0.05). In this statistical model, year and extraction solvent were nested within extraction timing with respect to AFEX pretreatment. **B** Linear regression of ethanol concentration and carbon dioxide concentration for all samples

**Fig. 7:**
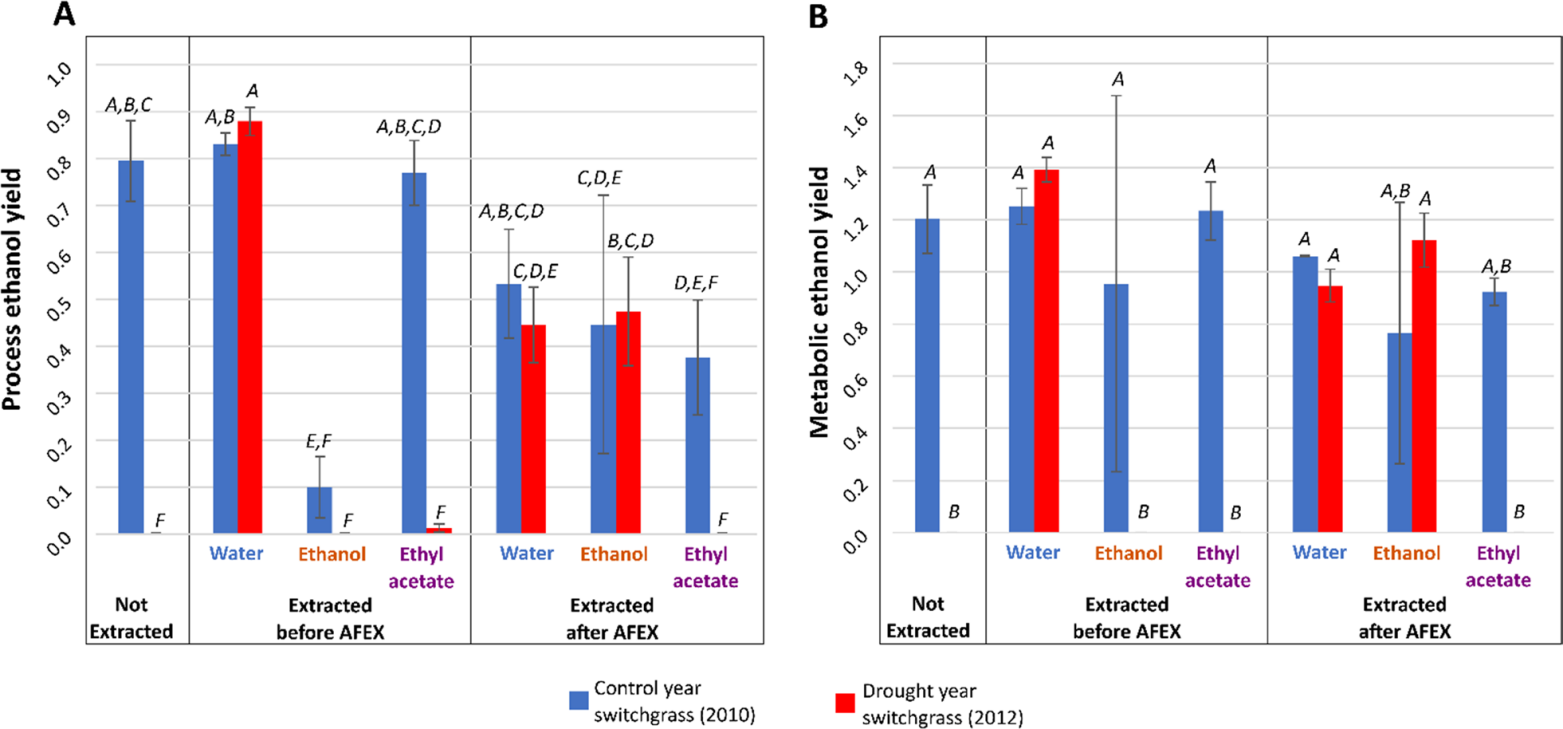
Ethanol yields reported to estimate the fermentation performance of engineered yeast. **A** Process ethanol yield represents the actual yield compared to the theoretical maximum from complete conversion of glucose and xylose in the hydrolysate to ethanol. This assumes a theoretical maximum conversion of 0.51 g ethanol/g sugar in the hydrolysate. **B** The metabolic ethanol yield represents the efficiency of the microbes at converting consumed sugars into fuel. This assumes a theoretical maximum conversion of 0.51 g ethanol/g consumed sugars. Samples that do not share a letter on top of the bars are significantly different based on Tukey’s pairwise statistical comparison (⍺ = 0.05). In this statistical model, year and extraction solvent were nested within extraction timing with respect to AFEX pretreatment

When evaluated based on final ethanol concentration, the drought-year, water-extracted switchgrass before AFEX-pretreatment generated the maximum amount of ethanol (Fig. 6A; Additional file 1: Tables S5, S6), followed closely by the water-extracted, untreated control-year switchgrass. The ethanol production results showed a significant correlation with the CO_2_ production data reported from the fermentation experiments (Fig. 6B). A single outlier was removed for a specific replicate of control-year switchgrass extracted with ethanol after AFEX-treatment due to inaccurate measurement of ethanol at the end of the experiment through HPLC analysis. Water-extracted drought-year switchgrass had the highest process and metabolic ethanol yield, though this was not statistically greater than some of the other samples (Fig. 7A, B; Additional file 1: Table S7A, B).

### Highest amounts of saponins were present in water extracts from drought-year switchgrass

Water, ethanol, and ethyl acetate extracts from untreated and AFEX-treated samples were analyzed for potential inhibitors using various mass spectrometry techniques. Accelerated solvent extractor (Dionex 350, Thermo Scientific) was used with an inbuilt program for the three solvents. Samples were extracted at 1600 psi and temperatures of 100 °C, 70 °C, and 77 °C for water, ethanol, and ethyl acetate, respectively. Inorganics, nitrogenous molecules, sugar acids, large hydrophilic molecules, polyphenols, glycerides, and non-structural sugars were removed using water (Additional file 1: Table S8A). Ethyl acetate and ethanol solvents targeted secondary metabolites, such as phenolic glycosides, alkaloids, flavonoids, tannins, and aromatic molecules [30, 41]. Ethyl acetate has previously been used to extract phenolic compounds generated during the AFEX process [17, 31] and a few of these compounds were present in ethyl acetate extracts from drought-stressed switchgrass (Additional file 1: Table S8C). Extracts for the three solvents were analysed for biomass compounds commonly listed as inhibitory toward yeast (Additional file 1: Table S8A–C). However, these compounds were present in comparable amounts in both control- and drought-year switchgrass, indicating they were less likely to be responsible for the observed yeast inhibition.

**Fig. 8:**
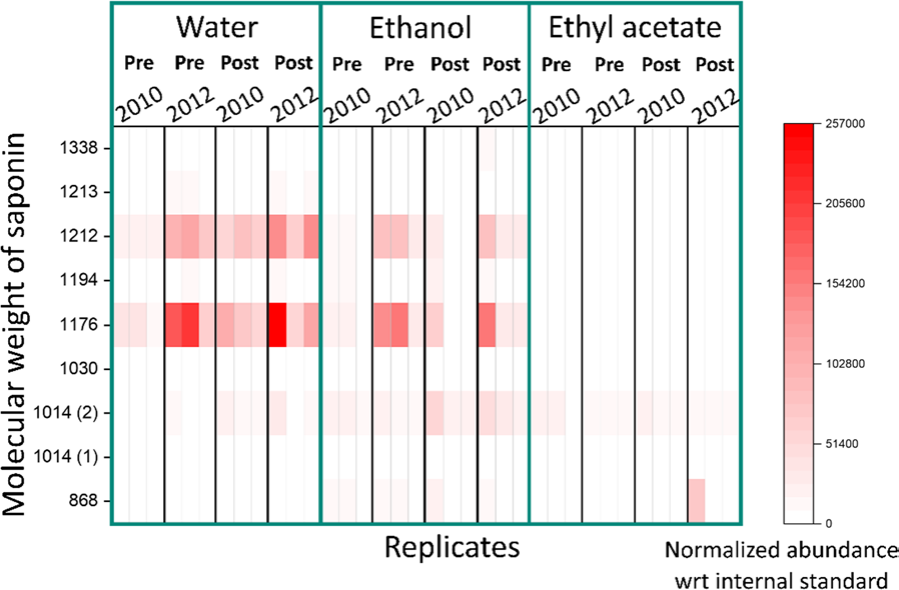
Normalized abundance of saponins with molecular weights 1176 and 1212 were higher in drought-year switchgrass (2012) to control year (2010) in water and ethanol extracts (*n* = 3). ’Pre’ stands for samples that were extracted then AFEX-pretreated while ‘Post’ for samples that were AFEX-pretreated then extracted. Values in ‘()’ represent the isomer for the saponin and ‘wrt’ stands for ‘with respect to’

Using non-targeted mass spectrometry, we identified a number of saponins with different molecular weights that were present in the water, ethanol, and ethyl acetate extracts before and after AFEX pretreatment. Non-targeted mass spectrometry detects both known and unknown compounds that are present in the extracts unlike the targeted approach that detects the presence of compounds known a priori. The amounts of each compound were reported as normalised abundances with respect to an internal standard—telmisartan. Of the compounds detected, a number of compounds annotated as saponins were present in higher abundance in the untreated drought-year water extracts (Fig. 8). The presence of saponins in crops such as agave, yucca and quillaja bark have led to yeast inhibition during ethanol production [42, 43] and could possibly contribute toward yeast inhibition in drought-stressed switchgrass.

The saponins with molecular weights 1176 and 1212 Da were in relatively greater abundance in drought year than the control switchgrass for water and ethanol extracts (Fig. 8; Additional file 1: Tables SS9A, B, S10A, B). However, it is difficult to determine if more saponins were extracted before or after AFEX (Fig. 8). Despite the presence of saponins in the ethanol extracts, the inhibitory nature of the ethanol extracted hydrolysates indicates possible inefficiency of saponin removal by ethanol to a level feasible for yeast growth, or extraction of other essential compounds necessary for the survival of yeast. Less-glycosylated saponins of lower molecular weights 868 and 1014 (isomer 2) Da were more prevalent in the ethyl acetate extracts (Fig. 8; Additional file 1: Table S11A, B). On comparing all the saponins extracted for the three solvents, water was relatively more efficient in extracting the higher molecular weight saponins from switchgrass than ethanol or ethyl acetate (Fig. 8), perhaps owing to more extensive glycosylation.

**Fig. 9:**
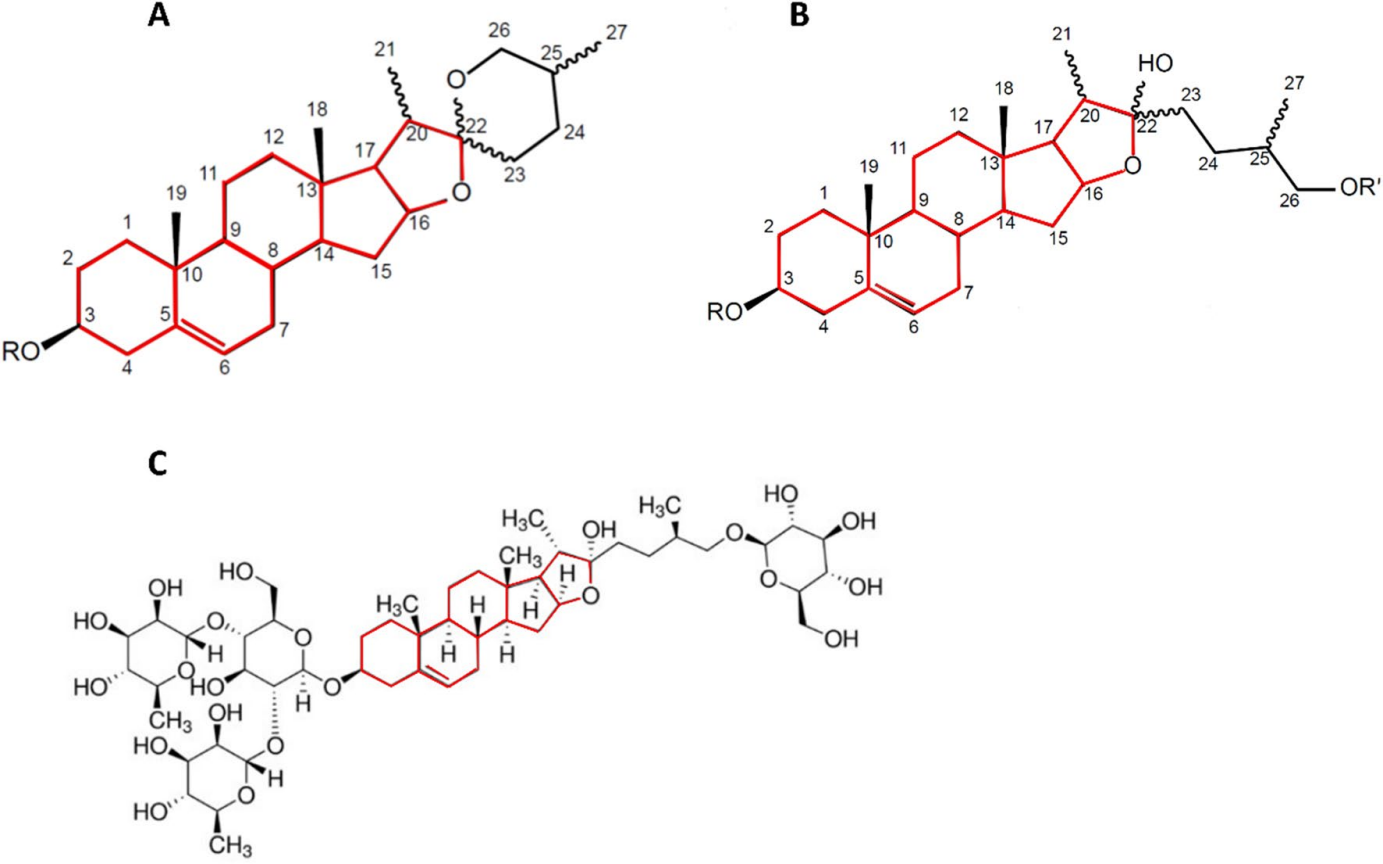
Molecular structure of proposed diosgenin derived aglycones for *m/z* 415 Da fragment ion for (**A**) a closed ring structure with side-chain glycosylation at the C-3 position [28], (**B**) an open ring structure with side-chain glycosylation at the C-3 and C-26 positions [28], and **C** protodioscin with the core aglycone ‘disogenin’ highlighted in red

Most of the annotated saponins had molecular weights higher than commercially available saponins, such as protodioscin (MW: 1049.2 g/mol) and soyasaponin (MW: 943.12 g/mol). Switchgrass saponins are also difficult to purify in the laboratory in large quantities [28]. The commercially available saponin protodioscin (Fig. 9C) shares the same aglycone structure (diosgenin) as the saponins detected in switchgrass water extracts (Fig. 9A). The diosgenin structure can have a closed ring at the 22-position (Fig. 9A) or an open ring (Fig. 9B) as in protodioscin (Fig. 9C), which is glycosylated with one glucose and two rhamnose units at the 3-position and one glucose at the 26-position [44]. Based on the molecular weight, we hypothesize that the 1176 saponin is glycosylated with three deoxyhexoses (e.g., rhamnose) and two hexoses (e.g., glucose or galactose) at the 3-position, with a closed ring diosgenin aglycone (Fig. 9A). There is some disagreement in literature as to whether the 1176 Da saponin (detected at *m/z* value of 1177 in positive mode MS) has a closed or open ring structure. Lee et al., proposed an open ring diosgenin aglycone with three rhamnoses and one glucose at the 3-position and one glucose at the 26-position, indicating it was dehydrated during MS analysis [16]. This is in contrast to Li et al. who proposed a closed structure for the same saponin (and no dehydration) [28]. The saponin with molecular weight 1212 Da may be glycosylated at 3-position and 26-position with two rhamnoses and three glucose units, similar to the protodioscin structure, with an additional glucose molecule. However, the exact arrangement of this molecule is unknown. More extensive mass spectrometry and NMR experiments would be needed to fully characterize these saponins, and is a subject for future research. Because of the similarities with our saponin structure, protodioscin was used to spike non-inhibitory switchgrass hydrolysates harvested in the control year (2010) and estimate the inhibitory nature of the saponin on yeast growth.

**Fig. 10:**
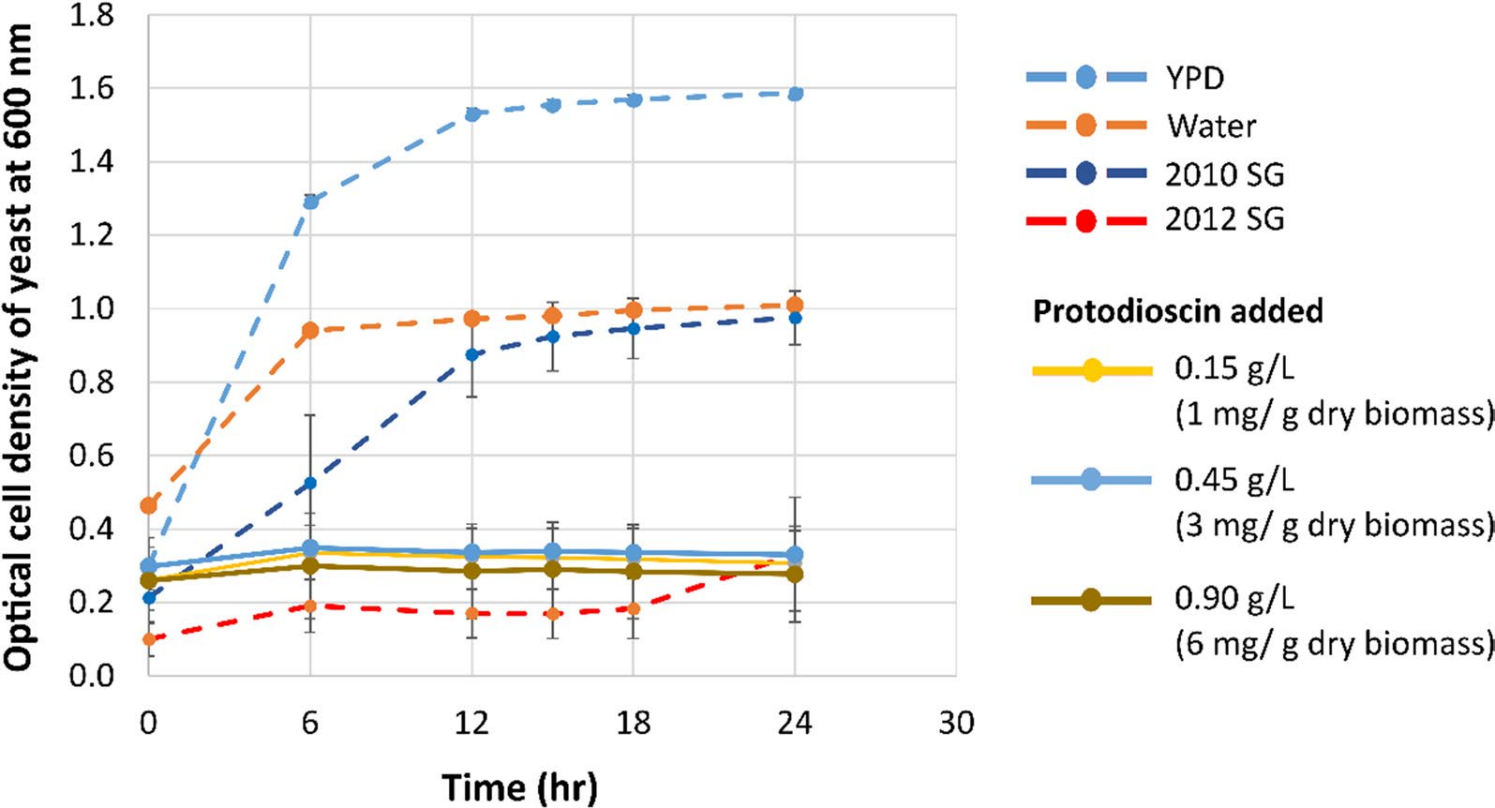
Yeast cell growth inhibited in 2012 switchgrass hydrolysate and in the presence of exogenous saponins added to the 2010 switchgrass hydrolysate. All growth curves were quantified using optical density of the media at 600 nm using a microplate reader. ‘()’ depicts the amount of additional saponin added to the hydrolysate per gram dry biomass loaded during enzymatic hydrolysis. ‘SG’ stands for switchgrass. Data points represent the average ± standard deviation (*n* = 3)

We evaluated the specific effect of protodioscin on yeast fermentation by adding protodioscin to control hydrolysate at concentrations previously reported in switchgrass. Various researchers have reported the highest concentration of saponins in switchgrass leaf blades compared to other tissues, ~ 3 mg saponins/g dry biomass (DB) [16, 28]. As stems make up the majority of switchgrass mass, it is expected that the actual concentration would be lower in a year with normal precipitation. Unextracted control-year (2010) and drought-year (2012) switchgrass hydrolysates replicated the yeast growth trend (Fig. 10) as observed in previously reported fermentation experiments (Fig. 4A) [17] with drought-year more inhibitory than the control. Protodioscin concentrations of 1, 3, and 6 mg/g dry biomass (DB) were added to 2010 switchgrass hydrolysates to determine the level of added saponin required to inhibit the yeast. All the protodioscin additions showed an inhibitory effect on the yeast cells after 24 h of fermentation in the microplate (Fig. 10) when compared to 2010 switchgrass hydrolysate that contained no added protodioscin. The slight increase in cell growth for all samples in the first 6 h of fermentation could be attributed to the presence of additional YPD media in the starting liquid inoculum. Unfortunately, it was not possible to load the wells using a cell pellet and achieve consistent results (data not shown). Even though the final optical densities were similar for water and 2010 switchgrass hydrolysate, the growth rate was slower in the 2010 switchgrass hydrolysate, and the additional media was insufficient to recover growth in the 2012 switchgrass hydrolysate (Fig. 10). The experiment serves as a proof of concept demonstrating the inhibitory effect of additional saponins added to hydrolysate from control (non-inhibitory) sample.

## Discussion

In spite of the higher sugar content in hydrolysate obtained from water-extraction of the pretreated biomass, the highest final concentration of ethanol was obtained in drought-year switchgrass that was extracted with water before AFEX-pretreatment (Figs. 4B, 7A). This can be attributed to efficient removal of yeast inhibitors using water compared to the other solvents used in this study. The slower fermentation rate in drought-year switchgrass that was water-extracted after AFEX (Fig. 4C) could be because the extraction process was less effective in extracting the inhibitory compounds after the pretreatment or due to removal of nutrients essential for the survival of yeast in the fermentation hydrolysate. Washing the biomass with distilled autoclaved water after extraction ensured complete removal of the extraction solvents—ethanol and ethyl acetate, which would be otherwise highly toxic to yeast growth. The lack of yeast growth in ethanol-extracted switchgrass before AFEX (Fig. 4D) and, thereby, low to no ethanol production (Fig. 6A) may be caused by the removal of essential nutrients required for yeast survival, although there was no evidence of this based on free amino acid analysis (Fig. 5; Additional file 1: Table S4A). The ethanol-extracted pretreated switchgrass showed a slower growth rate with a longer lag phase for the drought-year sample when compared to the paired control-year sample (Fig. 4E) with a moderate amount of ethanol production (Fig. 6A; Additional file 1: Tables S5, S6). Ethyl acetate was specifically chosen to extract phenolic inhibitory compounds present in the switchgrass generated during AFEX [19, 45, 46]. However, the ethyl acetate extraction showed no benefit on fermentation (Fig. 4F, G) and comparatively low removal of phenolic and acidic inhibitors (Additional file 1: Table SS8C) when compared to water extracts (Additional file 1: Table S8A).

Ammonia fiber expansion pretreatment is a completely dry process, does not include output liquid streams, and leaves minimal residual pretreatment chemicals in the biomass [47, 48]. Hence, a traditional solvent extraction step is never included for this method unlike in dilute acid or alkali pretreatment methods [34, 45]. Although all the soluble compounds remain in AFEX-treated biomass, prior research has shown that the washing or detoxification are typically not required prior to enzymatic hydrolysis and fermentation [32, 49, 50]. However, based on our results, it may be necessary to extract drought-stressed switchgrass with water before AFEX-pretreatment, something that was not necessary for the control-year switchgrass. Water extraction of the untreated drought-year switchgrass was most effective in overcoming microbial inhibition while achieving a greater ethanol concentration at the end of fermentation. The metabolic ethanol yield data also support this claim (Fig. 7B).

LC–MS analysis of the water extracts showed the presence of various saponins in comparatively higher amounts in drought-year water extracts than control-year water extracts (Additional file 1: Table S9A, B). Saponins are triterpene or steroidal glycosides and are commonly known as natural laundry detergents due to their ability to function as a surfactant [16]. Saponins with molecular weights 1176 and 1212 Da are expected to be steroidal saponins and were present in relatively higher amounts in the drought-year water extracts when compared to other saponins (Fig. 8). A previous study also reported that saponin 1176 and 1212 Da are present in greater amounts in upland switchgrass ecotypes, similar to the one used in this study, than lowland ecotypes [28]. In another study, saponin 1176 (*m/z*:1177) was characterized in detail using MS and NMR to determine glycosylation patterns [16]. Saponins have previously been recovered from switchgrass stems and leaves in concentrations ranging from 0.7 to 8.4 mg/g biomass, with greater amounts in leaf tissues of upland switchgrass ecotypes than lowland [16, 28]; however, based on MS of water-extracted switchgrass used in this project, we expect as much as 16-fold more saponins in the drought-stressed switchgrass (Additional file 1: Table S9A, B). This greater concentration of saponins due to drought stress has been seen with other crops [51–53]. *Panax ginseng* flower buds produced six unknown and four previously identified ginseng saponins commonly known as ginsenosides [54]. Olive trees that experienced drought when grown throughout the Mediterranean region accumulated saponins in their leaves [9]. Saponins are also abundant in desert plants, such as *Quillaja saponaria* and *Yucca schidigera*, which are used as commercial sources for saponin standards [55, 56].

Others have also demonstrated the cytotoxicity of saponins toward various microorganisms. Ibrahim et al., showed adverse effects of saponins obtained from *Sapindus mukorossi* and *Rheum emodi* on gram-positive bacteria [57]. Alcazar et al. reported cytotoxicity in yeast strains *Saccharomyces cerevisiae* and *Kluyveromyces marxianus by* steroidal saponins obtained from Agave, a desert plant [42]. Due to their toxic nature toward eukaryotic cells [16, 54, 58, 59], saponins may be potentially responsible for the yeast inhibition in fermentation hydrolysates obtained from drought-stressed switchgrass.

Water had the tendency to remove higher molecular weight saponins and, ethanol and ethyl acetate extracted more of the lower molecular weight saponins. This could possibly be caused due to the higher water solubility of more extensively glycosylated forms. The saponins with molecular weights 1176 and 1212 Da share the same core aglycone called diosgenin but differ in their sugar moieties [16, 28]. The presence of a relatively higher amount of saponins in water extracts obtained from untreated switchgrass corroborates with the fermentation profiles of water extracted untreated switchgrass (Fig. 4B). Saponins appear to withstand AFEX pretreatment (Fig. 8) and likely contribute toward microbial inhibition in unextracted drought-year switchgrass. Microplate fermentation experiments with various concentrations of protodioscin, a commercially available saponin, in control-year non-inhibitory hydrolysate demonstrated the ability of saponins to inhibit yeast growth (Fig. 10). LC–MS/MS analysis will be conducted on the compounds identified in water extracts in further experiments using more targeted MS-based approaches.

## Conclusions

This study overcame the challenge of microbial inhibition experienced by drought-stressed switchgrass to produce lignocellulosic ethanol by *S. cerevisiae*. Including an additional water extraction step before AFEX-pretreatment for drought-stressed switchgrass produced a comparable quantity of ethanol relative to the paired control-year samples and better than the unextracted control-year samples. Non-targeted LC–MS qualitative characterization of compounds showed that saponins, a class of naturally generated triterpene or steroidal glycosides, are more abundant in drought-stressed switchgrass and could potentially be responsible for the inhibition displayed by an engineered *S. cerevisiae* strain in drought-stress switchgrass hydrolysates.

In future work, the switchgrass extracts will be further concentrated and fractionated using solid-phase extraction. The fractions will be used in add-back fermentation experiments coupled with LC–MS/MS to test the extent of inhibition of *S. cerevisiae* and identify the critical fermentation inhibitor(s) with targeted MS^2^ approaches.

## Methods

### Feedstock production and composition analysis

Shawnee switchgrass, an upland cultivar, was produced at the Arlington Agricultural Research Station (ARL, 43° 17′ 45″ N, 89° 22′ 48″ W, 315 masl) in Arlington, Wisconsin. Switchgrass was grown on the plot ARL-346 but was harvested in different years (2010 and 2012). The Plano-silt-loam soil type (fine-silty, mixed, superactive, mesic Type Argiudoll); a deep (> 1 m), well-drained mollisol developed over glacial till and formed under tallgrass prairie dominated this region [16, 60]. Ambient growth conditions were described by 6.9 °C of mean annual temperature and 869 mm of average precipitation [61]. The methods for cultivation and nutrient supply are the same as previously reported [17, 62]. Switchgrass was harvested and chopped into a trailer. A representative sample was collected, milled through a 5 mm screen, and stored in plastic bags at ambient conditions until used. Switchgrass harvested in 2010 contained 34.24% glucan, 21.54% xylan, 14.90% total extractives and 17.87% Klason lignin (acid-insoluble lignin). Switchgrass harvested in the drought year (2012) composed 29.36% glucan, 18.91% xylan, 22.14% total extractives and 14.31% Klason lignin. Total biomass composition for the two switchgrass types used in this paper was the same as previously reported by Ong et al. [17] (Additional file 1: Table S1).

### AFEX-pretreatment

Ammonia fiber expansion pretreatment is a completely dry process that uses anhydrous ammonia to disrupt the cell wall to enable enzymes to access the carbohydrates. Custom-made 200 mL stainless-steel tubular reactors rated to 2000 psi were used to pretreat 25 g of switchgrass on dry biomass basis that was lightly packed in the reactor. Unextracted or solvent-extracted switchgrass from drought year (2012) and control year (2010) was adjusted to 60% moisture loading per gram dry biomass prior to pretreatment. The reactor was preheated to 95 °C and loaded with 2 g of anhydrous ammonia per gram of dry biomass using Harvard Apparatus’ HA33 syringe pump. The temperature was ramped to 120 ± 5 °C, at which point, the reaction time was initiated. At the end of 30 min, heating was stopped, and ammonia was vented rapidly inside a well-ventilated walk-in fume hood [63]. The pretreated switchgrass was removed and dried overnight in a custom drying box with laminar airflow to prevent microbial contamination during drying. The dried biomass was bagged in airtight Ziploc bags until further use.

### Solvent extraction

Untreated and AFEX-pretreated switchgrass were solvent extracted using a Dionex ASE 350 Accelerated Solvent Extractor (Thermo Scientific). Samples were placed in 100 mL stainless steel extraction cells and extracted at 1600 psi and 100 °C, 70 °C, and 77 °C for water, ethanol, and ethyl acetate, respectively [12, 19, 64]. The cells were heated for about 5 min to reach the target extraction temperature, followed by 7 min of static time to achieve maximum extraction. Three extraction cycles of 100 mL rinse volumes were used to extract compounds selectively using a single extraction cell. The extracted biomass was washed thrice, using 100 mL of room temperature distilled autoclaved water in each wash, to remove any residual solvent from the extraction process [31]. Washed biomass was dried in a custom-designed laminar airflow drying box for 3–6 days until the moisture content was less than 11%. Dried biomass was bagged in airtight Ziploc bags. AFEX pretreatment was then performed, as described previously, on solvent-extracted untreated biomass before enzymatic hydrolysis.

### Production of switchgrass hydrolysate

High solids loading enzymatic hydrolysis was performed with 7% glucan loading on each sample for 72 h. Glucan content for the extracted biomass was estimated using mass balances, assuming the solvents did not extract glucan. The sample loading was calculated based on this value to maintain 7% glucan loading for the hydrolysis experiments. Cellulase enzyme Novozyme 22257 and hemicellulase enzyme Novozyme 22244 (Novozymes, Franklinton NC, USA) were desalted using a disposable column (PD-10, Cytiva, VWR 95017-001) and protein concentration was determined using the Pierce™ BCA Protein Assay Kit (Pierce Biotechnology). An enzyme cocktail containing 70% cellulase and 30% hemicellulase by volume was used with a total loading of 25 mg protein per g glucan in the hydrolysate. Monobasic and dibasic potassium phosphates were used to prepare a 1 M stock buffer solution. Phosphate buffer at pH 3.0 and 0.1 M concentration was used to maintain hydrolysis pH between 4.5 and 5.2. Make-up water was added to reach the desired total working volume. Geneticin at a concentration of 12.5 µg/mL was used as an antibiotic to prevent contamination. Hydrolysates were sampled at 0, 24, 48, and 72 h to analyze sugar, alcohol, and acid content. The starting pH of the fermentation hydrolysate was maintained between 5.0 and 5.2 using 12 M hydrochloric acid or 10 M sodium hydroxide. The hydrolysates were sterile filtered using 0.2 µm Stericups^©^ to separate liquid hydrolysates from solids, which were discarded. The sterile filtered liquid hydrolysates were stored at − 20 °C until further use. Two of the replicates (water extracted and ethyl acetate extracted 2010 AFEX switchgrass) experienced contamination during the experiment and were not included in the reported results. A previous study optimized the conditions for high solids loading for AFEX pretreated switchgrass at 7% glucan loading in the hydrolysate [37]. The glucan loading was adjusted to 7% for all extracted samples using mass balances based on the starting glucan composition of each feedstock and the amount of material extracted from each sample. The glucan conversion and glucose yield were calculated using the following formulae.

$${\text{Glucan conversion}}\,({\text{g glucose released per g glucose in dry biomass}})=\frac{{{\text{diff in glucose mesured by HPLC}} \left(\frac{g}{L}\right) {\text{at}} \ 72 \ {\text{hr and}} \ 0 \ {\text{hr}}}}{{1000 \times {\text{glucan loading}}{ \% }\ ({\text{g glucan}}/{\text{mL}})}} \times \frac{{\text{MW}}_{\text{Glucan}}(162.14\frac{{\text{g}}}{{\text{mol}}})}{{\text{MW}}_{\text{Glucose}}(180.16\frac{{\text{g}}}{{\text{mol}}})}$$

$${\text{Glucose yield}}\,({\text{mg glucose released per g glucose in dry biomass}})=\frac{{ {\text{diff in glucose measured by HPLC}} \,({\text{g}}/{\text{L}})\, {\text{at 72 h and 0 h}} }}{{1000 \times {\text{glucan loading}}\,{\% }\,({\text{g glucan}}/ {\text{mL}})}} \times \frac{{{\text{MW}}_{\text{Glucan}}(162.14 \,{\text{g}}/{\text{mol}})}}{{{\text{MW}}_{\text{Glucose}}(180.16\,{\text{g}}/{\text{mol}})}} \times {\text{Volume of hydrolysate produced}} ({\text{mL}})$$

### Fermentation of switchgrass hydrolysate

Fermentations of switchgrass hydrolysates into ethanol by yeast were performed as previously described [33] with modifications. Sterile serum bottles were aliquoted with 4 mL of switchgrass hydrolysate. The *Saccharomyces cerevisiae* strain GLBRCY945, which was derived from GLBRCY560 [65] and contains an additional *flo8* deletion mutation was used for fermentation testing. GLBRCY945 yeast were cultured overnight in YPD (10 g/L yeast extract, 20 g/L peptone, 20 g/L dextrose) and diluted in YPD until the culture reached an OD_600_ (optical density at 600 nm wavelength) of 0.5 to 1.0. The yeast culture was subsequently centrifuged, resuspended in sterile distilled water and inoculated at an OD_600_ of 0.2 into sterile 60 mL Wheaton serum bottles containing 4 mL of specific switchgrass hydrolysates. Inoculated serum bottles were sealed with blue butyl 20 mm rubber caps (Chemglass Life Sciences) and then sparged with N_2_ gas to render the cultures anaerobic. The bottles were shaken on a platform at 120 rpm in a growth chamber set at 30 °C. The serum bottles were connected to respirometer cartridges using BD PrecisionGlide 23GX1 (0.6 mm × 25 mm) sterile needles pierced through the butyl caps. The volume of carbon dioxide generated during the fermentation experiment was quantified using the respirometer (AER-800; Challenge Technology; Springdale, AR, USA) for 45 h. Samples were centrifuged, and supernatants were analyzed at the end of the experiment using high-performance liquid chromatography (HPLC) coupled with refractive index detection (RID) to evaluate sugar and ethanol content [66]. A Beckman DU720 spectrophotometer was used to measure the final background-subtracted cell density (OD_600_). All experiments were performed in triplicates spread across three different fermentation batches to accommodate biological variability. Moreover, each drought-year sample was paired with the control-year sample to achieve this.

### Amino acid analysis

Hydrolysate samples were sent to Creative Proteomics for analysis of 18 different amino acids. Free amino acids were quantitatively analyzed using AB SCIEX API 4000 mass spectrometry (with positive mode electro-spray ionization) connected with a Waters Acquity UPLC. Standards for 21 amino acids were dissolved in 0.1N HCl to prepare a stocking solution of 2.5 µmol/mL. Standards were mixed together and diluted in 0.1% formic acid in water to obtain gradient concentrations from 0.01 nmol/mL to 20 nmol/mL. UPLC–MS/MS was injected with 10 µL of the standards for analysis. Ice-cold methanol with 300 µL was mixed with 100 µL of each sample in a 2 mL tube and vortexed for 1 min. The mixture was centrifuged at 12,000 rpm (17,709×*g*) and 4 °C for 10 min. The supernatant was passed through 0.22 µm membrane filter into another tube. 10 µL of the prepared sample was injected into the UPLC–MS/MS for the analysis. Water Acquity UPLC HSS T3 column (2.1 × 150 mm 1.8 µm) coupled with a VanGuard precolumn (2.1 × 5 mm 1.8 µm). Mobile phase A consisted pure water with 0.1% formic acid and mobile phase B consisted of acetonitrile with 0.1% formic acid. The column temperature was held at 25 °C with sample chamber temperature at 8 °C. The flow rate was maintained at 0.2 mL/min. The elution gradient was set at (time (min), (%A/%B)): 0(100/0), 8(100/0), 10(90/10), 20(90/10), 20.5(10/90), 22.5(10/90), 23(100/0), 27(100/0).

### Non-targeted characterization of liquid chromatography–mass spectrometry (LC–MS)

Extracts were diluted in 1:10 acetonitrile containing 0.1 µM telmisartan as an internal standard. Aliquots of 50 µL were mixed with 50 µL of MilliQ water to improve chromatographic performance, and the solutions were transferred to glass autosampler vials for analysis. Extracts were analyzed using liquid chromatography/mass spectrometry (LC/MS) on a Xevo G2-XS mass spectrometer interfaced to an Acquity I-class UPLC system and model 2777 autosampler (Waters Corp., Milford, MA USA). Separations were performed using a BEH C18 column (2.1 × 100 mm, 1.7 µm particles, Waters) held at 40 °C using linear gradient elution based on solvent A = 10 mM aqueous ammonium formate and solvent B = acetonitrile programmed as follows (time (min), %A/%B): 0.0 (99/1), 1.0 (99/1), 15.0 (1/99), 18.0 (1/99); 18.01 (99/1), 20.0 (99/1) at a total flow rate of 0.40 mL/min, with injection of 10 µL of extract. Mass spectra were acquired over the range of *m/z* 80–1500 using electrospray ionization in positive-ion continuum mode and extended dynamic range. Quasi-simultaneous of low-energy (6 V) and high-energy (ramp from 15 to 80 V) spectra (MS^E^ acquisition, using argon as collision gas) was performed using a scan time of 0.2 s/function with the acquisition of spectra of leucine enkephalin (lock mass reference) sampled every 10 s, but real-time mass correction was not applied. Separate injections were made with analysis in negative-ion mode, with all other parameters remaining the same. All instrument control was managed using MassLynx v 4.2 software (Waters Corp.).

### LC–MS data processing

Raw MassLynx data files were imported into Progenesis QI software (v 2.4; Waters Corp.). Default parameters were used for thresholding, and Progenesis software performed peak detection, chromatographic retention time alignment, mass correction using leucine enkephalin lock mass reference, peak integration, isotopic, and adduct deconvolution, and normalization to the signal from the telmisartan internal standard. To aid annotation, experimental compound masses were used to calculate relative mass defect (RMD) values which reflect the fractional hydrogen content of each [67, 68]. Metabolite annotations were performed using a combination of manual spectrum interpretation supported by searches of multiple ChemSpider spectrum databases with a mass tolerance of 10 ppm.

### Microplate fermentation experiment

Seed culture was prepared by inoculating 6 individual colonies of *Saccharomyces cerevisiae* (GLBRCY945) from a freshly prepared agar plate in 100 mL of YPD media (10 g/L yeast extract and 20 g/L peptone was autoclaved, and 75 g/L dextrose solution was passed through 0.2 µm PES filter) in a shake flask for 12 h. Inoculum volume of seed culture was calculated to target OD of 7.4, so that the final microplate OD of ~ 0.1 was reached. Cells were centrifuged at 4000 rpm (3220×*g*) for 5 min at 22 °C. The supernatant was removed, and cell pellet was resuspended in freshly prepared YPD media (10 g/L yeast extract and 20 g/L peptone was autoclaved, and 20 g/L dextrose solution (0.2 µm sterile-filtered)). Fermentation experiments were performed in a flat-bottom 96-well plate with a well volume of 300 µL. A working volume was maintained at 200 µL with three technical replicates for each sample. Blank samples with no inoculum were present for each media. Eppendorf Research® plus 8-channel mechanical pipette was used to inoculate 65 µL of seed culture at the same time for each replicate. Sterile transparent microplate sealing film was used to seal the microplate for fermentation. Each well was pierced with 2 tiny holes that were diagonally opposite at the edge of each well to prevent carbon dioxide build-up and formation of large condensation bubbles over the course of fermentation. The microplate was placed inside a VWR® Barnstead static incubator at a temperature of 30 °C throughout the course of the experiment. The absorbance of the samples was quantified at 600 nm over 24 h using a microplate reader (Epoch 238451, BioTek Instruments, Inc.). YPD (10 g/L yeast extract and 20 g/L peptone was autoclaved, and 20 g/L dextrose solution (0.2 µm sterile-filtered)) was used as positive control and distilled autoclaved water was used as a negative control for *S. cerevisiae* growth.

## Supplementary Information

**Additional file 1.** Supplemental methods and data: biomass composition, hydrolysate composition, and statistical analyses.
