# Supplementary material for: Water-soluble saponins accumulate in drought-stressed switchgrass and may inhibit yeast growth during bioethanol production
Source: Biotechnol Biofuels Bioprod. 2022 Oct 31;15:116. doi: 10.1186/s13068-022-02213-y (PMC9620613; doi:10.1186/s13068-022-02213-y)
Supplement: Supplementary file 1 — Additional file 1. Supplemental methods and data: biomass composition, hydrolysate composition, and statistical analyses. [file 13068_2022_2213_MOESM1_ESM.docx]

**Supporting Information**

Water-soluble saponins accumulate in drought-stressed switchgrass and may inhibit yeast growth during bioethanol production

Sarvada Chipkar^1,3^, Katie Smith^1,3^, Elizabeth M Whelan^2^, Derek J Debrauske^2^, Annie Jen^2,6^, Katherine A. Overmyer^6,8^, Andrea Senyk^1,3^, Larkin Hooker-Moericke^1,3^, Marissa Gallmeyer^1,3^, Joshua J. Coon^6,7,8^, A. Daniel Jones^4,5^, Trey K. Sato^2^, Rebecca G. Ong^1,3^

^1^Department of Chemical Engineering, Michigan Technological University

^2^ DOE Great Lakes Bioenergy Research Center, University of Wisconsin-Madison

^3^DOE Great Lakes Bioenergy Research Center, Michigan Technological University

^4^ RTSF Mass Spectrometry & Metabolomics Core, Michigan State University

^5^ Department of Biochemistry and Molecular Biology, Michigan State University

^6^ Department of Biomolecular Chemistry, University of Wisconsin-Madison

^7^ Department of Chemistry, University of Wisconsin–Madison

^8^ Morgridge Institute for Research, Madison, WI

Correspondence: [rgong1@mtu.edu](mailto:rgong1@mtu.edu) (Rebecca G. Ong)

**Contents:**

- Method for estimating composition (% dry biomass) of solvent-extracted switchgrass on small scale.
- Method for analyzing inhibitory compounds in switchgrass water extracts using LC-MS/MS
- Table 1. Switchgrass composition (% dry biomass) harvested in 2010 and 2012
- Table 2. Composition (% dry biomass) of extracted switchgrass harvested in 2010 and 2012
- Table 3A. General linear regression for glucan conversion for enzymatically hydrolyzed switchgrass after 72 hours
- Table 3B. General linear regression for glucose yield for enzymatically hydrolyzed switchgrass after 72 hours
- Table 4A. Amino acid composition in hydrolysates of ethanol extracted switchgrass
- Table 4B. Amino acid composition in hydrolysates of water extracted switchgrass
- Figure 1. Amino acid content in hydrolysates
- Table 5. Ethanol and carbon dioxide data for respirometer fermentation experiments
- Table 6. General linear regression for final ethanol yield for fermented switchgrass
- Table 7A. General linear regression for process ethanol yield for fermented switchgrass
- Table 7B. General linear regression for metabolic ethanol yield for fermented switchgrass
- Table 8A. Inhibitory compounds composition in water extracts
- Table 8B. Inhibitory compounds composition in ethanol extracts
- Table 8C. Inhibitory compounds composition in ethyl acetate extracts
- Table 9A. Normalized saponin abundance in water extracts of switchgrass extracted before AFEX treatment for paired control and drought-year samples
- Table 9B. Normalized saponin abundance in water extracts of switchgrass extracted after AFEX treatment for paired control and drought-year samples
- Table 10A. Normalized saponin abundance in ethanol extracts of switchgrass extracted before AFEX treatment for paired control and drought-year samples
- Table 10B. Normalized saponin abundance in ethanol extracts of switchgrass extracted after AFEX treatment for paired control and drought-year samples
- Table 11A. Normalized saponin abundance in ethyl acetate extracts of switchgrass extracted before AFEX treatment for paired control and drought-year samples
- Table 11B. Normalized saponin abundance in ethyl acetate extracts of switchgrass extracted after AFEX treatment for paired control and drought-year samples

#### Method for estimating composition (% dry biomass) of solvent-extracted switchgrass on small scale.

NREL standard procedures[1-4] were used to analyze composition of solvent-extracted biomass based on deviations used by Ong et al, 2016 [5] with following variations. Water extracts and hydrolyzed water extracts were run through an Aminex HPX-87H column (Bio-Rad, Hercules, CA) equipped with a compatible guard column to analyze sucrose, glucose, fructose, xylose, cellobiose, arabinose, acetate, and formate based on calibration standards. Thermo’s Dionex Accelerated Solvent Extractor 350 was used for extraction using conditions mentioned in the NREL procedures. Ethanol extractives were quantified using Genevac Rocket^TM^ Evaporation System at a temperature of 42°C under vacuum for 3 hours. The acid hydrolysis on extracted biomass were performed in 21 mL pressure vials with a working volume of 17.64 mL. Whatman glass fiber filters were used for ashing hydrolyzed biomass. Thermo Scientific’s NanoDrop One spectrophotometer was used for acid-soluble lignin analysis.

#### Method for analyzing inhibitory compounds in switchgrass water extracts using LC-MS/MS

Sample extracts were removed from 5°C and maintained on ice throughout preparation. Each sample was diluted to 10-fold and 75-fold using chilled 2.5 µM 13C6 vanillin as diluent. Each sample dilution was centrifuged at 14,000 x g for 2 minutes to pellet potential particulates, then transferred to an amber glass autosampler vial with glass vial insert for analysis. A primary lignotoxin standard comprised of 84 different lignotoxins and phenolic compounds was prepared. A calibration curve was developed using 12 standards obtained from the primary lignotoxin standard by performing two-fold dilutions in the range of 0.024 µM to 50 µM. Liquid chromatography with tandem mass spectrometry (LC-MS/MS) was employed to analyze the samples. Five microliters of sample was injected into ACQUITY UPLC USS T3 reversed-phase column (Waters Corporation). A temperature of 40°C and flow rate of 150 µL/min was maintained throughout the analysis. Two different mobile phases were used for the analysis. Mobile phase A contained 0.1% formic acid in water, and mobile phase B contained 100% acetonitrile. Mobile phase B was increased from 7.5% to 30% for 20 minutes. It was increased to 100% in the next 14.5 minutes and held at this rate for the last 1.5 minutes. The column was re-equilibrated with mobile phase B at 7.5% for 2 minutes before injecting the next sample. The LC system was coupled to a Q Exactive Orbitrap mass spectrometer via heated electrospray ionization (HESI II) (Thermo Scientific). Source conditions were as follows: HESI II and capillary temperature at 275°C, sheath gas flow rate at 30 units, aux gas flow rate at 6 units, sweep gas flow rate at 0 units, spray voltage at |4.0k| for positive mode and |4.5k| for negative mode, and S-lens RF at 60.0 units. To promote fragmentation, in-source collision-induced dissociation (CID) energy was set to 25.0 eV. The MS was operated in a polarity switching mode acquiring positive and negative full MS and targeted MS2 spectra (top 2) within the same injection. Acquisition parameters for full MS scans in both modes were 35,000 resolution, 1 x 10^5^ automatic gain control (AGC) target, 50 ms ion accumulation time (max IT), and 50-360 m/z scan range. Targeted MS2 scans in both modes were then performed at 17,500 resolution, 1 x 10^5^ AGC target, 100 ms max IT, 1.0 m/z isolation window, stepped normalization collision energy (NCE) at 20, 30, 40, and a 5.0 s dynamic exclusion. The retention time window for each compound was optimized from prior experiments. The resulting LC-MS data was manually processed using a custom TraceFinder 4.1 (Thermo Scientific) method using a mass precision of 4 ppm and mass tolerance of 10 ppm. Peak area analysis used a prepared standard solution to locate and identify similar peaks. Results were exported into Microsoft Excel 2010, and statistics were summarised.

**Table 1. Switchgrass composition (% dry biomass) harvested in 2010 and 2012.** Data originally published in Ong et al., 2016. Data represents average ± standard deviation for n=3.

|  | | | **Switchgrass** | | | |
| --- | --- | --- | --- | --- | --- | --- |
| **Composition (%)** | | | **2010 (Control)** | | **2012 (Drought)** | |
| **Total Ash** | | | 5.56 ± 0.16 | | 5.47 ± 0.08 | |
| **Total Extractives** | | | 14.90 ± 0.26 | | 22.14 ± 0.21 | |
|  | **Water Extractives** | **Sucrose** | | 0.43 ± 0.01 | | 2.40 ± 0.07 |
|  |  | **Fructose** | | 0.51 ± 0.01 | | 2.33 ± 0.02 |
|  |  | **Glucose** | | 0.62 ± 0.01 | | 2.57 ± 0.03 |
|  |  | **Xylose** | | ND | | 0.04 ± 0.00 |
|  |  | **Galactose** | | ND | | 0.06 ± 0.00 |
|  |  | **Arabinose** | | ND | | 0.04 ± 0.00 |
|  |  | **Mannose** | | NM | | ND |
|  |  | **Glucan** | | 0.62 ± 0.02 | | 0.98 ± 0.38 |
|  |  | **Xylan** | | 0.06 ± 0.00 | | 0.07 ± 0.01 |
|  |  | **Galactan** | | 0.05 ± 0.00 | | 0.31 ± 0.02 |
|  |  | **Arabinan** | | 0.18 ± 0.01 | | 0.07 ± 0.01 |
|  |  | **Mannan** | | NM | | ND |
|  | **Ethanol Extractives** | | 2.08 ± 0.19 | | 2.61 ± 0.03 | |
|  | **Extracted Protein** | | 1.16 ± 0.09 | | 1.89 ± 0.41 | |
| **Total Protein** | | | 3.19 ± 0.09 | | 4.65 ± 0.08 | |
| **Total Structural Sugars**^‡^ | | | 59.50 ± 0.18 | | 52.28 ± 0.09 | |
|  | **Glucan** | | 34.24 ± 0.11 | | 29.36 ± 0.07 | |
|  | **Xylan** | | 21.54 ± 0.13 | | 18.91 ± 0.04 | |
|  | **Galactan** | | 1.43 ± 0.03 | | 1.31 ± 0.02 | |
|  | **Arabinan** | | 2.30 ± 0.03 | | 2.69 ± 0.01 | |
|  | **Mannan** | | NM | | ND | |
|  | **Acetyl Groups** | | 2.58 ± 0.11 | | 2.15 ± 0.06 | |
| **Total Lignin** | | | 18.59 ± 0.11 | | 15.38 ± 0.06 | |
|  | **Acid Insoluble Lignin** | | 17.87 ± 0.10 | | 14.31 ± 0.06 | |
|  | **Acid Soluble Lignin** | | 0.73 ± 0.03 | | 1.07 ± 0.01 | |
| **Mass Balance Closure** | | | 102.44 | | 100.17 | |

*NM = not measured

^†^ND = not detected

^‡^Total structural sugars does not include acetyl groups

**Table 2. Composition (% dry biomass) of extracted switchgrass harvested in 2010 and 2012.** ‘Avg’ stands for average, ‘SD’ for standard deviation for n=3 calculated using error propagation and ‘NM’ for not measured.

| Feedstock Composition (%) | Water extracted switchgrass | | | | Ethanol extracted switchgrass | | | | Ethyl acetate extracted switchgrass | | | |
| --- | --- | --- | --- | --- | --- | --- | --- | --- | --- | --- | --- | --- |
|  | 2010 (Control) | | 2012 (Drought) | | 2010 (Control) | | 2012 (Drought) | | 2010 (Control) | | 2012 (Drought) | |
|  | Avg | SD | Avg | SD | Avg | SD | Avg | SD | Avg | SD | Avg | SD |
| **Total extractives** | 9.16 | 0.57 | 11.02 | 0.91 | 10.75 | 2.71 | 10.32 | 0.88 | 12.08 | 0.89 | 15.39 | 2.01 |
| Water extractives |  |  |  |  |  |  |  |  |  |  |  |  |
| Sucrose | 0.01 | 0.01 | 0 | 0.01 | 0.20 | 0.35 | 0.18 | 0.05 | 0.49 | 1.34 | 0.21 | 0.04 |
| Fructose | 0 | 0.01 | 0 | 0 | 0 | 0 | 0.33 | 0.03 | 0.35 | 0.26 | 0.92 | 0.07 |
| Glucose | 0 | 0 | 1.59 | 1.09 | 0 | 0 | 0.27 | 0.03 | 0.72 | 0.80 | 0.60 | 0.05 |
| Xylose | NM |  | NM |  | NM |  | NM |  | NM |  | NM |  |
| Galactose | NM |  | NM |  | NM |  | NM |  | NM |  | NM |  |
| Arabinose | NM |  | NM |  | NM |  | NM |  | NM |  | NM |  |
| Mannose | NM |  | NM |  | NM |  | NM |  | NM |  | NM |  |
| Glucan | 0.35 | 0.03 | 0.04 | 0.21 | 0.88 | 0.56 | 0.67 | 0.35 | 0 | 0 | 0.42 | 0.03 |
| Xylan | 0.10 | 0.01 | 0.13 | 0.01 | 0.29 | 0.37 | 0.47 | 0.10 | 0.31 | 0.01 | 0.68 | 0.16 |
| Arabinan | 0.03 | 0.00 | 0.03 | 0.00 | 0.05 | 0.05 | 0.07 | 0.02 | 0.05 | 0.00 | 0.08 | 0.01 |
| Mannan | NM |  | NM |  | NM |  | NM |  | NM |  | NM |  |
| Ethanol extractives | 6.57 | 2.04 | 5.70 | 2.46 | 6.69 | 3.83 | 3.22 | 1.08 | 5.12 | 1.97 | 3.51 | 2.59 |
| **Total structural sugars** |  |  |  |  |  |  |  |  |  |  |  |  |
| Glucan | 29.02 | 3.57 | 29.47 | 2.88 | 31.56 | 3.34 | 29.45 | 2.69 | 27.90 | 4.88 | 26.54 | 3.55 |
| Xylan | 13.90 | 3.98 | 17.72 | 3.96 | 18.14 | 1.91 | 18.33 | 2.37 | 13.37 | 4.31 | 15.29 | 4.47 |
| Galactan | NM |  | NM |  | NM |  | NM |  | NM |  | NM |  |
| Arabinan | 1.80 | 0.27 | 2.34 | 0.38 | 2.02 | 0.15 | 2.41 | 0.28 | 1.75 | 0.38 | 2.13 | 0.51 |
| Mannan | NM |  | NM |  | NM |  | NM |  | NM |  | NM |  |
| Acetyl groups | 2.18 | 0.25 | 2.38 | 0.17 | 2.06 | 0.32 | 2.48 | 0.37 | 2.21 | 0.31 | 2.37 | 0.38 |
| **Total Lignin** | 33.43 | 8.62 | 24.31 | 3.24 | 19.97 | 5.80 | 22.86 | 9.18 | 33.70 | 6.37 | 25.50 | 6.94 |
| Acid insoluble lignin | 31.70 | 8.62 | 22.68 | 3.23 | 18..74 | 5.80 | 21.39 | 9.17 | 32.07 | 6.36 | 23.73 | 6.93 |
| Acid soluble lignin | 1.73 | 0.26 | 1.63 | 0.18 | 1.24 | 0.26 | 1.47 | 0.14 | 1.63 | 0.21 | 1.76 | 0.37 |
| **Mass Balance closure** | 89.49 | | 87.23 | | 84.51 | | 85.85 | | 91.01 | | 87.22 | |

**Table 3A. General linear regression for glucan conversion for enzymatically hydrolyzed switchgrass after 72 hours.** Samples with different letters are significantly different based on Tukey’s pairwise statistical comparison (⍺ = 0.05).

**Analysis of Variance**

| **Source** | **DF** | **Adj SS** | **Adj MS** | **F-Value** | **P-Value** |
| --- | --- | --- | --- | --- | --- |
| Year | 1 | 0.01340 | 0.013401 | 7.42 | 0.011 |
| Extraction solvent (Year) | 6 | 0.03200 | 0.005333 | 2.95 | 0.025 |
| Extraction type wrt AFEX(Year, Extraction solvent) | 6 | 0.13183 | 0.021972 | 12.16 | 0.000 |
| Error | 26 | 0.04698 | 0.001807 |  |  |
| Total | 39 | 0.22846 |  |  |  |

**Tukey Pairwise Comparisons: Extraction type wrt AFEX(Year, Extraction solvent). Grouping Information Using the Tukey Method and 95% Confidence**

| **Extraction type wrt AFEX(Year, Extraction solvent)** | **N** | **Mean** | **Grouping** | | | | |
| --- | --- | --- | --- | --- | --- | --- | --- |
| Post(2012, Water) | 3 | 0.642112 | A |  |  |  |  |
| Post(2010, Water) | 2 | 0.613696 | A | B |  |  |  |
| Post(2012, Ethyl acetate) | 3 | 0.608424 | A | B |  |  |  |
| Post(2010, Ethyl acetate) | 2 | 0.602094 | A | B | C |  |  |
| Post(2012, Ethanol) | 3 | 0.560106 | A | B | C | D |  |
| Pre(2012, Water) | 3 | 0.516474 | A | B | C | D | E |
| None(2012, None) | 3 | 0.494947 |  | B | C | D | E |
| Post(2010, Ethanol) | 3 | 0.487368 |  | B | C | D | E |
| Pre(2012, Ethanol) | 3 | 0.485337 |  | B | C | D | E |
| Pre(2010, Ethanol) | 3 | 0.483003 |  | B | C | D | E |
| Pre(2012, Ethyl acetate) | 3 | 0.467382 |  |  | C | D | E |
| Pre(2010, Water) | 3 | 0.455542 |  |  |  | D | E |
| None(2010, None) | 3 | 0.450977 |  |  |  | D | E |
| Pre(2010, Ethyl acetate) | 3 | 0.419788 |  |  |  |  | E |

*Means that do not share a letter are significantly different.*

**Table 3B. General linear regression for glucose yield for enzymatically hydrolyzed switchgrass after 72 hours.** Samples with different letters are significantly different based on Tukey’s pairwise statistical comparison (⍺ = 0.05).

**Analysis of Variance**

| **Source** | **DF** | **Adj SS** | **Adj MS** | **F-Value** | **P-Value** |
| --- | --- | --- | --- | --- | --- |
| Year | 1 | 548.0 | 548.03 | 13.80 | 0.001 |
| Extraction solvent(Year) | 6 | 3367.5 | 561.25 | 14.14 | 0.000 |
| Extraction type wrt AFEX(Year, Extraction solvent) | 6 | 3437.5 | 572.92 | 14.43 | 0.000 |
| Error | 26 | 1032.3 | 39.71 |  |  |
| Total | 39 | 8405.4 |  |  |  |

**Tukey Pairwise Comparisons: Extraction type wrt AFEX(Year, Extraction solvent). Grouping Information Using the Tukey Method and 95% Confidence**

| **Extraction type wrt AFEX(Year, Extraction solvent)** | **N** | **Mean** | **Grouping** | | | |
| --- | --- | --- | --- | --- | --- | --- |
| Post(2012, Water) | 3 | 85.6207 | A |  |  |  |
| Post(2012, Ethyl acetate) | 3 | 80.0348 | A |  |  |  |
| Post(2010, Water) | 2 | 76.7120 | A | B |  |  |
| Post(2010, Ethyl acetate) | 2 | 73.8037 | A | B | C |  |
| Post(2012, Ethanol) | 3 | 72.8138 | A | B |  |  |
| Pre(2012, Water) | 3 | 66.9690 | A | B | C |  |
| Pre(2012, Ethyl acetate) | 3 | 59.6564 |  | B | C | D |
| Pre(2010, Water) | 3 | 58.4549 |  | B | C | D |
| Post(2010, Ethanol) | 3 | 57.6363 |  | B | C | D |
| Pre(2010, Ethyl acetate) | 3 | 57.3308 |  | B | C | D |
| None(2012, None) | 3 | 52.8275 |  |  | C | D |
| Pre(2012, Ethanol) | 3 | 43.6803 |  |  |  | D |
| Pre(2010, Ethanol) | 3 | 43.6354 |  |  |  | D |
| None(2010, None) | 3 | 42.4598 |  |  |  | D |

*Means that do not share a letter are significantly different.*

**Table 4A. Amino acid composition in hydrolysates of ethanol extracted switchgrass:** Mean values for amino acids (nmol/ml) obtained using Tukey's pairwise comparison with 95% confidence interval for unextracted and ethanol extracted hydrolysates before and after AFEX-treatment. Superscripts with different alphabets represent statistically different values for a specific amino acid in a treatment type with an alpha value of 0.15.

|  | Hydrolysate Amino Acid Concentration (nmol/mL) in ethanol extracted switchgrass | | | | | | | | |
| --- | --- | --- | --- | --- | --- | --- | --- | --- | --- |
| Treatment Type | Solvent type nested within extraction timing | | | Harvest year interaction with extraction timing | | | | | |
| Amino acid | Unextracted (n=6) | Untreated (n=6) | AFEX-treated (n=6) | Unextracted (n=3) | | Untreated (n=3) | | AFEX-treated (n=3) | |
|  |  |  |  | 2010 (Control) | 2012 (Drought) | 2010 (Control) | 2012 (Drought) | 2010 (Control) | 2012 (Drought) |
| Asparagine | 55^A^ | 17^B^ | 1^C^ | 67^A^ | 43^B^ | 18^C^ | 17^C^ | 2^D^ | 1^D^ |
| Aspartic acid | 58^A^ | 57^A^ | 1^B^ | 64^A^ | 51^B^ | 64^A^ | 51^B^ | 2^C^ | 1^C^ |
| Arginine | 26^A^ | 13^B^ | 4^C^ | 34^A^ | 18^B^ | 15^B^ | 12^B,C^ | 4^C^ | 3^C^ |
| Lysine | 20^A^ | 17^A^ | 15^A^ | 13^A^ | 18^A^ | 20^A^ | 20^A^ | 18^A^ | 17^A^ |
| Glutamine | 6^A^ | 6^A^ | 26^A^ | 5^A^ | 7^A^ | 6^A^ | 6^A^ | 40^A^ | 11^A^ |
| Proline | 669^A^ | 72^B^ | 240^B^ | 259^B^ | 1079^A^ | 64^C^ | 79^C^ | 232^B^ | 249^B^ |
| Tyrosine | 78^B^ | 71^B^ | 17^A^ | 71^A^ | 86^A^ | 75^A^ | 67^A^ | 128^A^ | 106^A^ |
| Phenylalanine | 66^A^ | 53^A^ | 85^A^ | 48^A^ | 83^A^ | 57^A^ | 49^A^ | 106^A^ | 64^A^ |
| Alanine | 357^A^ | 136^A^ | 293^B^ | 224^B,C^ | 490^A^ | 138^C^ | 134^C^ | 199^B,C^ | 388^A,B^ |
| Glycine | 119^A^ | 110^A^ | 11^B^ | 113^A^ | 125^A^ | 111^A^ | 109^A^ | 4^B^ | 17^B^ |
| Serine | 59^A^ | 27^B^ | 2^C^ | 34^B^ | 84^A^ | 29^B^ | 25^B^ | 1^C^ | 3^C^ |
| Glutamic acid | 114^A^ | 160^A^ | 58^B^ | 101^A,B,C^ | 126^A,B,C^ | 174^A^ | 146^A,B^ | 71^B,C^ | 45^C^ |
| Isoleucine | 56^A^ | 35^A,B^ | 17^B^ | 33^B,C^ | 80^A^ | 38^B^ | 32^B,C^ | 13^C^ | 21^B,C^ |
| Leucine | 143^A^ | 131^A^ | 96^A^ | 111^A^ | 174^A^ | 136^A^ | 126^A^ | 106^A^ | 87^A^ |
| Threonine | 191^A^ | 104^B^ | 37^C^ | 139^B^ | 243^A^ | 104^C^ | 104^C^ | 37^D^ | 39^D^ |
| Valine | 181^A^ | 102^A^ | 102^A^ | 98^B,C^ | 265^A^ | 114^B,C^ | 91^B,D^ | 130^A,B^ | 73^B,D^ |
| Histidine | 9^B^ | 6^B^ | 27^A^ | 4^D^ | 13^B^ | 4^D^ | 7^C,D^ | 13^B,C^ | 42^A^ |
| Tryptophan | 9^A^ | 3^B^ | 8^A,B^ | 6^B,C^ | 13^A^ | 3^C^ | 2^C^ | 10^A,B^ | 5^B,C^ |

| Hydrolysate Amino Acid Concentration (nmol/mL) in ethanol extracted switchgrass | | | | |
| --- | --- | --- | --- | --- |
| Treatment Type | Harvest year interaction with solvent type | | | |
| Amino acid | Unextracted (n=3) | | Ethanol (n=6) | |
|  | 2010 (Control) | 2012 (Drought) | 2010 (Control) | 2012 (Drought) |
| Asparagine | 67^A^ | 43^B^ | 10^C^ | 9^C^ |
| Aspartic acid | 64^A^ | 51^B^ | 32^C^ | 26^C^ |
| Arginine | 34^A^ | 18^B^ | 10^C^ | 7^C^ |
| Lysine | 13^A^ | 18^A^ | 19^A^ | 18^A^ |
| Glutamine | 5^A^ | 7^A^ | 23^A^ | 9^A^ |
| Proline | 259^B^ | 1079^A^ | 148^C^ | 164^C^ |
| Tyrosine | 71^A^ | 86^A^ | 101^A^ | 86^A^ |
| Phenylalanine | 48^A^ | 83^A^ | 82^A^ | 57^A^ |
| Alanine | 224^B^ | 490^A^ | 168^B^ | 261^B^ |
| Glycine | 113^A^ | 125^A^ | 57^B^ | 63^B^ |
| Serine | 34^B^ | 84^A^ | 15^C^ | 14^C^ |
| Glutamic acid | 101^A^ | 126^A^ | 122^A^ | 96^A^ |
| Isoleucine | 33^B^ | 80^A^ | 26^B^ | 27^B^ |
| Leucine | 111^A^ | 174^A^ | 121^A^ | 106^A^ |
| Threonine | 139^B^ | 243^A^ | 70^C^ | 72^C^ |
| Valine | 98^B,C^ | 265^A^ | 122^B^ | 82^B,C^ |
| Histidine | 4^D^ | 13^B^ | 9^C^ | 25^A^ |
| Tryptophan | 6^B,C^ | 13^A^ | 7^B^ | 4^B,D^ |

**Table 4B. Amino acid composition in hydrolysates of water extracted switchgrass:** Mean values for amino acids (nmol/ml) obtained using Tukey's pairwise comparison with 95% confidence interval for unextracted and water extracted hydrolysates before and after AFEX-treatment. Superscripts with different alphabets represent statistically different values for a specific amino acid in a treatment type with an alpha value of 0.15.

|  | Hydrolysate Amino Acid Concentration (nmol/mL) in water extracted switchgrass | | | | | | | | |
| --- | --- | --- | --- | --- | --- | --- | --- | --- | --- |
| Treatment Type | Solvent type nested within extraction timing | | | Harvest year interaction with extraction timing | | | | | |
| Amino acid | Unextracted (n=6) | Untreated (n=6) | AFEX-treated (n=6) | Unextracted (n=3) | | Untreated (n=3) | | AFEX-treated | |
|  |  |  |  | 2010 (Control) | 2012 (Drought) | 2010 (Control) | 2012 (Drought) | 2010 (Control) (n=2) | 2012 (Drought) (n=3) |
| Asparagine | 55^A^ | 12^B^ | 7^B^ | 67^A^ | 43^B^ | 15^C^ | 9^C^ | 13^C^ | 1^C^ |
| Aspartic acid | 58^A^ | 23^B^ | 4^B^ | 64^A^ | 51^A,B^ | 12^D^ | 34^B,C^ | 11^C,D^ | 0^D^ |
| Arginine | 26^A^ | 12^A^ | 15^A^ | 34^A^ | 18^A,B^ | 12^A,B^ | 13^A,B^ | 25^A,B^ | 6^B^ |
| Lysine | 15^B^ | 14^B^ | 36^A^ | 13^B^ | 18^B^ | 13^B^ | 14^B^ | 49^A^ | 22^B^ |
| Glutamine | 6^B^ | 5^B^ | 16^A^ | 5^B^ | 7^B^ | 4^B^ | 5^B^ | 26^A^ | 6^B^ |
| Proline | 669^A^ | 33^C^ | 125^B^ | 259^B^ | 1079^A^ | 22^C^ | 43^C^ | 104^C^ | 145^B,C^ |
| Tyrosine | 78^A^ | 69^A^ | 119^A^ | 71^A^ | 86^A^ | 61^A^ | 76^A^ | 146^A^ | 92^A^ |
| Phenylalanine | 66^A^ | 43^A^ | 60^A^ | 48^A^ | 83^A^ | 38^A^ | 47^A^ | 71^A^ | 49^A^ |
| Alanine | 357^A^ | 141^B^ | 262^A,B^ | 224^B^ | 490^A^ | 101^B^ | 181^B^ | 233^B^ | 290^A,B^ |
| Glycine | 119^A^ | 117^A^ | 20^B^ | 113^A^ | 125^A^ | 100^A^ | 134^A^ | 23^B^ | 17^B^ |
| Serine | 59^A^ | 22^B^ | 3^C^ | 34^B^ | 84^A^ | 19^C,D^ | 24^B,C^ | 5^D,E^ | 1^E^ |
| Glutamic acid | 114^A^ | 82^A^ | 34^B^ | 101^A,B^ | 126^A^ | 66^B,D,E^ | 98^A,B^ | 67^B,C,D^ | 12^C,E^ |
| Isoleucine | 56^A^ | 24^A^ | 30^A^ | 33^B^ | 80^A^ | 21^B^ | 27^B^ | 52^A,B^ | 17^B^ |
| Leucine | 143^A^ | 108^A^ | 99^A^ | 110^A^ | 174^A^ | 95^A^ | 120^A^ | 150^A^ | 66^A^ |
| Threonine | 191^A^ | 83^B^ | 41^B^ | 139^B^ | 243^A^ | 74^C^ | 92^C^ | 72^C^ | 26^D^ |
| Valine | 181^A^ | 62^B^ | 78^A,B^ | 98^B^ | 265^A^ | 49^B^ | 75^B^ | 123^B^ | 56^B^ |
| Histidine | 9^A^ | 3^B^ | 25^B^ | 4^C^ | 13^B^ | 2^C^ | 3^C^ | 17^B^ | 32^A^ |
| Tryptophan | 9^A,B^ | 2^B^ | 13^A^ | 6^A^ | 13^A^ | 2^A^ | 2^A^ | 17^A^ | 10^A^ |

| Hydrolysate Amino Acid Concentration (nmol/mL) in water extracted switchgrass | | | | |
| --- | --- | --- | --- | --- |
| Treatment Type | Harvest year interaction with solvent type | | | |
| Amino acid | Unextracted (n=3) | | Water | |
|  | 2010 (Control) | 2012 (Drought) | 2010 (Control) (n=5) | 2012 (Drought) (n=6) |
| Asparagine | 67^A^ | 43^B^ | 14^C^ | 5^C^ |
| Aspartic acid | 64^A^ | 51^A^ | 11^B^ | 17^B^ |
| Arginine | 34^A^ | 18^A,B^ | 19^A,B^ | 10^B^ |
| Lysine | 13^B^ | 18^B^ | 31^A^ | 18^A,B^ |
| Glutamine | 5^B^ | 7^B^ | 15^A^ | 5^B^ |
| Proline | 259^B^ | 1079^A^ | 63^C^ | 94^C^ |
| Tyrosine | 71^A^ | 86^A^ | 104^A^ | 84^A^ |
| Phenylalanine | 48^A^ | 83^A^ | 54^A^ | 48^A^ |
| Alanine | 224^B^ | 490^A^ | 167^B^ | 236^B^ |
| Glycine | 113^A^ | 125^A^ | 61^B^ | 76^B^ |
| Serine | 34^B^ | 84^A^ | 12^C^ | 13^C^ |
| Glutamic acid | 101^A,B^ | 126^A^ | 67^B,C^ | 56^C^ |
| Isoleucine | 33^B^ | 80^A^ | 36^B^ | 22^B^ |
| Leucine | 110^A^ | 174^A^ | 122^A^ | 93^A^ |
| Threonine | 139^B^ | 243^A^ | 73^C^ | 59^C^ |
| Valine | 98^B^ | 265^A^ | 86^B^ | 66^B^ |
| Histidine | 4^C^ | 13^A,B^ | 10^B,C^ | 18^A^ |
| Tryptophan | 6^A^ | 13^A^ | 9^A^ | 6^A^ |

####
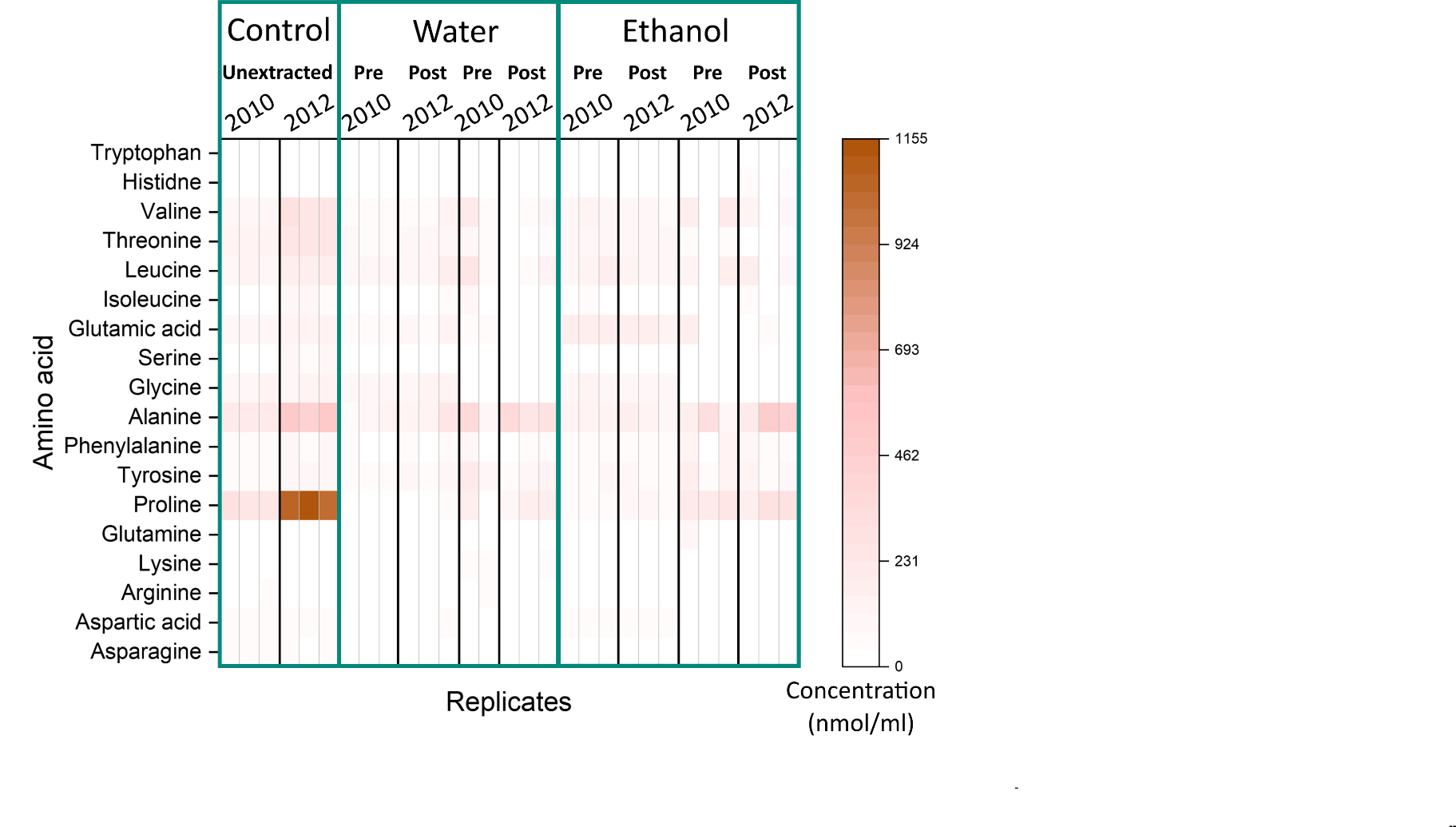


***Figure 1****. Proline was present in highest amounts in unextracted drought-year switchgrass for the18 different amino acids reported in unextracted and, water and ethanol extracted switchgrass hydrolysates. ’Pre’ stands for samples that were extracted then AFEX-pretreated while ‘Post’ for samples that were AFEX-pretreated then extracted.*

**Table 5. Ethanol and carbon dioxide data for respirometer fermentation experiments:** Final ethanol concentration (g/L) and carbon dioxide volume (ml) in respirometer experiments. ‘Avg’ stands for average; ‘Std Dev’ for standard deviation of the population.

| Compound | Ethanol (g/L) | | | | | | Carbon dioxide (mL) | | | | | |
| --- | --- | --- | --- | --- | --- | --- | --- | --- | --- | --- | --- | --- |
| Extraction type | 2010 (Control) | | | 2012 (Drought) | | | 2010 (Control) | | | 2012 (Drought) | | |
|  | Avg |  | Std Dev | Avg |  | Std Dev | Avg |  | Std Dev | Avg |  | Std Dev |
| Unextracted | 21.31 | ± | 0.63 | 0.06 | ± | 0.02 | 43.40 | ± | 1.05 | 1.55 | ± | 0.19 |
| Water extracted then AFEX-pretreated | 24.57 | ± | 0.99 | 30.23 | ± | 0.68 | 45.71 | ± | 2.06 | 55.98 | ± | 0.92 |
| AFEX-pretreated then water extracted | 20.31 | ± | 2.76 | 18.41 | ± | 2.90 | 39.50 | ± | 2.36 | 39.94 | ± | 4.41 |
| Ethanol extracted then AFEX-pretreated | 3.01 | ± | 1.96 | 0.05 | ± | 0.02 | 7.24 | ± | 4.60 | 1.46 | ± | 0.42 |
| AFEX-pretreated then ethanol extracted | 15.89 | ± | 9.75 | 19.14 | ± | 3.06 | 46.87 | ± | 1.75 | 40.86 | ± | 5.26 |
| Ethyl acetate extracted then AFEX-pretreated | 20.40 | ± | 0.59 | 0.34 | ± | 0.22 | 35.90 | ± | 4.88 | 1.41 | ± | 0.50 |
| AFEX-pretreated then ethyl acetate extracted | 15.15 | ± | 4.53 | 0.04 | ± | 0.02 | 30.88 | ± | 9.85 | 1.68 | ± | 0.07 |

**Table 6. General linear regression for final ethanol yield for fermented switchgrass**. Samples with different letters are significantly different based on Tukey’s pairwise statistical comparison (⍺ = 0.05).

**Analysis of Variance**

| **Source** | **DF** | **Adj SS** | **Adj MS** | **F-Value** | **P-Value** |
| --- | --- | --- | --- | --- | --- |
| Year | 1 | 774.0 | 773.96 | 48.70 | 0.000 |
| Extraction solvent(Year) | 6 | 2645.9 | 440.98 | 27.75 | 0.000 |
| Extraction type wrt AFEX(Year, Extraction solvent) | 6 | 1060.0 | 176.67 | 11.12 | 0.000 |
| Error | 26 | 413.2 | 15.89 |  |  |
| Total | 39 | 4704.7 |  |  |  |

**Tukey Pairwise Comparisons: Extraction type wrt AFEX(Year, Extraction solvent). Grouping Information Using the Tukey Method and 95% Confidence**

| **Extraction type wrt AFEX(Year, Extraction solvent)** | **N** | **Mean** | **Grouping** | | | |
| --- | --- | --- | --- | --- | --- | --- |
| Pre(2012, Water) | 3 | 30.2268 | A |  |  |  |
| Pre(2010, Water) | 3 | 24.5669 | A | B |  |  |
| None(2010, None) | 3 | 21.3058 | A | B |  |  |
| Pre(2010, Ethyl acetate) | 3 | 20.3967 | A | B |  |  |
| Post(2010, Water) | 2 | 20.3080 | A | B |  |  |
| Post(2012, Ethanol) | 3 | 19.1382 | A | B |  |  |
| Post(2012, Water) | 3 | 18.4149 | A | B |  |  |
| Post(2010, Ethanol) | 3 | 15.8930 |  | B |  |  |
| Post(2010, Ethyl acetate) | 2 | 15.1541 |  | B | C |  |
| Pre(2010, Ethanol) | 3 | 3.0073 |  |  | C | D |
| Pre(2012, Ethyl acetate) | 3 | 0.3377 |  |  |  | D |
| None(2012, None) | 3 | 0.0624 |  |  |  | D |
| Pre(2012, Ethanol) | 3 | 0.0457 |  |  |  | D |
| Post(2012, Ethyl acetate) | 3 | 0.0444 |  |  |  | D |

*Means that do not share a letter are significantly different.*

**Table 7A. General linear regression for process ethanol yield for fermented switchgrass.** Samples with different letters are significantly different based on Tukey’s pairwise statistical comparison (⍺ = 0.05).

**Analysis of Variance**

| **Source** | **DF** | **Adj SS** | **Adj MS** | **F-Value** | **P-Value** |
| --- | --- | --- | --- | --- | --- |
| Year | 1 | 1.1439 | 1.14386 | 74.90 | 0.000 |
| Extraction solvent(Year) | 6 | 2.2841 | 0.38068 | 24.93 | 0.000 |
| Extraction type wrt AFEX(Year, Extraction solvent) | 6 | 1.0931 | 0.18218 | 11.93 | 0.000 |
| Error | 26 | 0.3971 | 0.01527 |  |  |
| Total | 39 | 4.7417 |  |  |  |

**Tukey Pairwise Comparisons: Extraction type wrt AFEX(Year, Extraction solvent). Grouping Information Using the Tukey Method and 95% Confidence**

| **Extraction type wrt AFEX(Year, Extraction solvent)** | **N** | **Mean** | **Grouping** | | | | | |
| --- | --- | --- | --- | --- | --- | --- | --- | --- |
| Pre(2012, Water) | 3 | 0.879604 | A |  |  |  |  |  |
| Pre(2010, Water) | 3 | 0.831529 | A | B |  |  |  |  |
| None(2010, None) | 3 | 0.795722 | A | B | C |  |  |  |
| Pre(2010, Ethyl acetate) | 3 | 0.770021 | A | B | C | D |  |  |
| Post(2010, Water) | 2 | 0.533202 | A | B | C | D |  |  |
| Post(2012, Ethanol) | 3 | 0.474798 |  | B | C | D |  |  |
| Post(2010, Ethanol) | 3 | 0.447019 |  |  | C | D | E |  |
| Post(2012, Water) | 3 | 0.445423 |  |  | C | D | E |  |
| Post(2010, Ethyl acetate) | 2 | 0.376174 |  |  |  | D | E | F |
| Pre(2010, Ethanol) | 3 | 0.099685 |  |  |  |  | E | F |
| Pre(2012, Ethyl acetate) | 3 | 0.012663 |  |  |  |  |  | F |
| None(2012, None) | 3 | 0.002215 |  |  |  |  |  | F |
| Pre(2012, Ethanol) | 3 | 0.001379 |  |  |  |  |  | F |
| Post(2012, Ethyl acetate) | 3 | 0.001112 |  |  |  |  |  | F |

*Means that do not share a letter are significantly different.*

**Table 7B. General linear regression for metabolic ethanol yield for fermented switchgrass.** Samples with different letters are significantly different based on Tukey’s pairwise statistical comparison (⍺ = 0.05).

**Analysis of Variance**

| **Source** | **DF** | **Adj SS** | **Adj MS** | **F-Value** | **P-Value** |
| --- | --- | --- | --- | --- | --- |
| Year | 1 | 3.764 | 3.76392 | 39.58 | 0.000 |
| Extraction solvent(Year) | 6 | 5.314 | 0.88558 | 9.31 | 0.000 |
| Extraction type wrt AFEX(Year, Extraction solvent) | 6 | 2.399 | 0.39984 | 4.20 | 0.004 |
| Error | 26 | 2.473 | 0.09510 |  |  |
| Total | 39 | 13.442 |  |  |  |

**Tukey Pairwise Comparisons: Extraction type wrt AFEX(Year, Extraction solvent). Grouping Information Using the Tukey Method and 95% Confidence**

| **Extraction type wrt AFEX(Year, Extraction solvent)** | **N** | **Mean** | **Grouping** | |
| --- | --- | --- | --- | --- |
| Pre(2012, Water) | 3 | 1.39342 | A |  |
| Pre(2010, Water) | 3 | 1.25146 | A |  |
| Pre(2010, Ethyl acetate) | 3 | 1.23482 | A |  |
| None(2010, None) | 3 | 1.20236 | A |  |
| Post(2012, Ethanol) | 3 | 1.12182 | A |  |
| Post(2010, Water) | 2 | 1.06058 | A |  |
| Pre(2010, Ethanol) | 3 | 0.95487 | A |  |
| Post(2012, Water) | 3 | 0.94778 | A |  |
| Post(2010, Ethyl acetate) | 2 | 0.92383 | A | B |
| Post(2010, Ethanol) | 3 | 0.76572 | A | B |
| None(2012, None) | 3 | 0.00000 |  | B |
| Post(2012, Ethyl acetate) | 3 | 0.00000 |  | B |
| Pre(2012, Ethyl acetate) | 3 | 0.00000 |  | B |
| Pre(2012, Ethanol) | 3 | 0.00000 |  | B |

*Means that do not share a letter are significantly different.*

**Table 8A. Inhibitory compound composition in water extracts:** Lignotoxins analysis of control year and drought year switchgrass water extracts that were extracted before and after AFEX pretreatment

|  | Inhibitory compounds concentration in water extracts (µM) | | | | | | | | | | | |
| --- | --- | --- | --- | --- | --- | --- | --- | --- | --- | --- | --- | --- |
|  | AFEX-Pretreated | | | | | | Untreated | | | | | |
| Compound (in µM) | 2010 (Control) | | | 2012 (Drought) | | | 2010 (Control) | | | 2012 (Drought) | | |
|  | Avg |  | Std Dev | Avg |  | Std Dev | Avg |  | Std Dev | Avg |  | Std Dev |
| 2-Isopropylmalic Acid | 0.47 | ± | 0.01 | 0.44 | ± | 0.03 | 0.26 | ± | 0.26 | 0.46 | ± | 0.00 |
| 2-Pyrazine acetic Acid | 0.35 | ± | 0.25 | 1.93 | ± | 0.88 | - | ± | - | - | ± | - |
| 3,4-Dihydroxybenzaldehyde | - | ± | - | - | ± | - | 0.48 | ± | 0.24 | 0.60 | ± | 0.12 |
| 3,4-Dihydroxybenzoic Acid | 0.60 | ± | 0.27 | 0.62 | ± | 0.05 | 1.40 | ± | 0.83 | - | ± | - |
| 4-Hydroxbenzoic Acid | 3.86 | ± | 0.33 | 2.64 | ± | 0.13 | 2.14 | ± | 1.10 | 1.13 | ± | 0.03 |
| 4-Hydroxyacetophenone | - | ± | - | - | ± | - | 0.11 | ± | 0.11 | - | ± | - |
| 4-Hydroxybenzaldehyde | 11.64 | ± | 0.68 | 5.77 | ± | 1.83 | 7.32 | ± | 3.42 | 4.19 | ± | 0.18 |
| 4-Hydroxybenzamide | 1.92 | ± | 0.15 | 1.36 | ± | 0.18 | - | ± | - | - | ± | - |
| 4-Hydroxyphenyllactic Acid | 0.51 | ± | 0.02 | 0.56 | ± | 0.03 | - | ± | - | - | ± | - |
| Acetosyringone | 2.62 | ± | 0.70 | 2.22 | ± | 0.83 | - | ± | - | - | ± | - |
| Azelaic Acid | 7.91 | ± | 0.67 | 7.19 | ± | 1.67 | 9.13 | ± | 0.00 | 5.82 | ± | 0.00 |
| Benzamide | 2.84 | ± | 0.00 | 2.61 | ± | 0.16 | - | ± | - | - | ± | - |
| Caffeic Acid | - | ± | - | - | ± | - | 3.33 | ± | 1.48 | 8.53 | ± | 2.38 |
| Chlorogenic acid | - | ± | - | - | ± | - | 6.59 | ± | 3.37 | 24.26 | ± | 1.15 |
| Coniferyladehyde | - | ± | - | - | ± | - | 2.42 | ± | 0.81 | 1.65 | ± | 0.00 |
| Coumaryl Amide | - | ± | - | 365.34 | ± | 20.43 | - | ± | - | - | ± | - |
| Delta-valerlactam | 0.24 | ± | 0.08 | 0.50 | ± | 0.09 | - | ± | - | 0.04 | ± | 0.05 |
| Ferulic Acid | 12.58 | ± | 1.75 | 14.58 | ± | 0.00 | 5.55 | ± | 1.63 | 5.57 | ± | 0.59 |
| Feruloyl Amide | 273.38 | ± | 7.87 | 260.64 | ± | 50.40 | - | ± | - | - | ± | - |
| Gamma-valerlactone | - | ± | - | 2.19 | ± | 3.10 | - | ± | - | - | ± | - |
| Hydrocaffeic Acid | 0.64 | ± | 0.04 | 0.68 | ± | 0.05 | - | ± | - | - | ± | - |
| Kynurenic Acid | - | ± | - | 0.31 | ± | 0.06 | - | ± | - | 0.17 | ± | 0.12 |
| p-Coumaric Acid | 4.03 | ± | 0.46 | 2.90 | ± | 0.43 | 0.88 | ± | 0.11 | 0.57 | ± | 0.03 |
| Salicylic Acid | - | ± | - | 1.30 | ± | 0.00 | 0.18 | ± | 0.00 | - | ± | - |
| Sinapinaldehyde | - | ± | - | - | ± | - | 0.94 | ± | 1.33 | - | ± | - |
| Suberic Acid | 2.21 | ± | 0.33 | 1.15 | ± | 0.28 | 0.45 | ± | 0.00 | 0.86 | ± | 0.08 |
| Syringaldehyde | - | ± | - | - | ± | - | 1.35 | ± | 1.92 | 1.12 | ± | 1.58 |
| Syringamide | 4.27 | ± | 0.00 | 4.07 | ± | 0.00 | - | ± | - | - | ± | - |
| Vanillic Acid | 13.51 | ± | 0.49 | 5.12 | ± | 5.12 | 6.86 | ± | 6.86 | - | ± | - |
| Vanillin | - | ± | - | - | ± | - | 4.60 | ± | 0.00 | 0.13 | ± | 0.00 |
| Vanillyl alcohol | - | ± | - | 2.96 | ± | 0.20 | - | ± | - | - | ± | - |
| Vanillylamide | 12.69 | ± | 0.81 | 9.70 | ± | 1.56 | - | ± | - | - | ± | - |

The symbol “-“ indicates that the compound was not detected in that particular sample. ‘Avg’ stands for average; ‘Std Dev’ for standard deviation of the population for n=3.

**Table 8B. Inhibitory compound composition in ethanol extracts:** Lignotoxins analysis of control year and drought year switchgrass ethanol extracts that were extracted before and after AFEX pretreatment

|  | Inhibitory compounds concentration in ethanol extracts (µM) | | | | | | | | | | | |
| --- | --- | --- | --- | --- | --- | --- | --- | --- | --- | --- | --- | --- |
|  | AFEX-Pretreated | | | | | | Untreated | | | | | |
| Compound (in µM) | 2010 (Control) | | | 2012 (Drought) | | | 2010 (Control) | | | 2012 (Drought) | | |
|  | Avg |  | Std Dev | Avg |  | Std Dev | Avg |  | Std Dev | Avg |  | Std Dev |
| 2-Pyrazine acetic Acid | - | ± | - | 3.18 | ± | 0.81 | - | ± | - | - | ± | - |
| 3,4-Dihydroxybenzaldehyde | 0.45 | ± | - | 0.44 | ± | 0.15 | 0.52 | ± | 0.06 | 0.77 | ± | 0.02 |
| 3,4-Dihydroxybenzoic Acid | 0.54 | ± | 0.27 | 0.75 | ± | 0.18 | - | ± | - | 1.76 | ± | 0.00 |
| 4-Hydroxbenzoic Acid | 3.50 | ± | 0.31 | 2.64 | ± | 0.27 | 3.19 | ± | 0.12 | 1.23 | ± | 0.03 |
| 4-Hydroxyacetophenone | - | ± | - | 1.39 | ± | 0.13 | - | ± | - | - | ± | - |
| 4-hydroxybenzaldehyde | 29.79 | ± | 4.11 | 12.94 | ± | 0.69 | 12.10 | ± | 1.22 | 5.24 | ± | 0.28 |
| 4-Hydroxybenzamide | 1.87 | ± | 0.11 | 1.56 | ± | 0.06 | - | ± | - | - | ± | - |
| 4-Hydroxyphenyllactic Acid | - | ± | - | 0.65 | ± | 0.00 | - | ± | - | - | ± | - |
| 5-Hydroxymethylfurfural | - | ± | - | - | ± | - | - | ± | - | 1.20 | ± | 1.20 |
| Acetosyringone | 3.40 | ± | 0.16 | 4.30 | ± | 0.40 | - | ± | - | - | ± | - |
| Azelaic Acid | 6.62 | ± | 0.94 | 11.17 | ± | 0.63 | 6.56 | ± | 0.09 | 7.88 | ± | 0.31 |
| Benzamide | 2.47 | ± | 0.08 | 3.65 | ± | 0.13 | - | ± | - | - | ± | - |
| Caffeic Acid | 0.09 | ± | 0.13 | 0.22 | ± | 0.15 | 0.50 | ± | 0.06 | 0.86 | ± | 0.10 |
| Chlorogenic acid | - | ± | - | - | ± | - | 2.82 | ± | 2.06 | 39.85 | ± | 6.10 |
| Cinnamamide | - | ± | - | 0.14 | ± | 0.10 | - | ± | - | - | ± | - |
| Coniferyladehyde | - | ± | - | - | ± | - | 2.66 | ± | 0.25 | 1.81 | ± | 0.00 |
| Coumaryl Amide | - | ± | - | - | ± | - | - | ± | - | - | ± | - |
| Delta-valerlactam | 11.80 | ± | 1.92 | 9.77 | ± | 1.65 | 10.84 | ± | 2.10 | 11.08 | ± | 2.62 |
| Ferulic Acid | 15.55 | ± | 2.69 | 15.31 | ± | 2.37 | 4.84 | ± | 0.42 | 4.53 | ± | 0.20 |
| Feruloyl Amide | 279.15 | ± | 8.86 | 325.57 | ± | 45.37 | - | ± | - | - | ± | - |
| Hydrocaffeic Acid | 0.60 | ± | 0.03 | 0.74 | ± | 0.10 | - | ± | - | - | ± | - |
| Kynurenic Acid | - | ± | - | 0.17 | ± | 0.12 | - | ± | - | 0.08 | ± | 0.12 |
| p-Coumaric Acid | 3.18 | ± | 0.34 | 2.66 | ± | 0.29 | 1.52 | ± | 0.78 | 1.06 | ± | 0.19 |
| Salicylic Acid | - | ± | - | - | ± | - | - | ± | - | 1.20 | ± | 0.00 |
| Sebacic Acid | - | ± | - | - | ± | - | - | ± | - | - | ± | - |
| Sinapinaldehyde | - | ± | - | - | ± | - | 1.09 | ± | 1.09 | - | ± | - |
| Suberic Acid | 1.60 | ± | 0.03 | 1.36 | ± | 0.07 | 1.08 | ± | 0.34 | 0.85 | ± | 0.15 |
| Syringaldehyde | - | ± | - | - | ± | - | 4.09 | ± | 0.05 | 3.45 | ± | 0.00 |
| Syringamide | 5.12 | ± | 0.00 | - | ± | - | - | ± | - | - | ± | - |
| Vanillic Acid | 11.89 | ± | 1.64 | - | ± | - | 7.60 | ± | 5.43 | - | ± | - |
| Vanillin | 10.62 | ± | 0.00 | 5.59 | ± | 0.96 | 3.23 | ± | 0.46 | 1.46 | ± | 0.34 |
| Vanillyl alcohol | - | ± | - | 3.42 | ± | 0.118 | - | ± | - | - | ± | - |
| Vanillylamide | 12.005 | ± | 0.53 | 13.94 | ± | 2.306 | - | ± | - | - | ± | - |

The symbol “-“ indicates that the compound was not detected in that particular sample. ‘Avg’ stands for average; ‘Std Dev’ for standard deviation of the population for n=3.

**Table 8C**. **Inhibitory compound composition in ethyl acetate extracts:** Lignotoxins analysis of control year and drought year switchgrass ethyl acetate extracts that were extracted before and after AFEX pretreatment

|  | Inhibitory compounds concentration in ethyl acetate extracts (µM) | | | | | | | | | | | |
| --- | --- | --- | --- | --- | --- | --- | --- | --- | --- | --- | --- | --- |
|  | AFEX-Pretreated | | | | | | Untreated | | | | | |
| Compound (in µM) | 2010 (Control) | | | 2012 (Drought) | | | 2010 (Control) | | | 2012 (Drought) | | |
|  | Avg |  | Std Dev | Avg |  | Std Dev | Avg |  | Std Dev | Avg |  | Std Dev |
| 4-Hydroxyacetophenone | 0.38 | ± | 0.01 | 0.43 | ± | 0.05 | - | ± | - | - | ± | - |
| 4-hydroxybenzaldehyde | 6.99 | ± | 1.02 | 4.71 | ± | 0.10 | 4.70 | ± | 0.84 | 1.14 | ± | 1.61 |
| 4-Hydroxybenzamide | 0.21 | ± | 0.21 | 0.13 | ± | 0.18 | - | ± | - | - | ± | - |
| Acetosyringone | 0.36 | ± | 0.52 | - | ± | - | - | ± | - | - | ± | - |
| Coumaryl Amide | 58.68 | ± | 8.74 | 66.26 | ± | 42.10 | - | ± | - | - | ± | - |
| Delta-valerlactam | 14.88 | ± | 2.27 | 21.83 | ± | 4.32 | 17.14 | ± | 1.11 | 15.35 | ± | 2.44 |
| Ferulic Acid | - | ± | - | 3.75 | ± | 0.07 | - | ± | - | - | ± | - |
| Gamma-valerlactone | 3.70 | ± | 0.98 | 3.17 | ± | 0.88 | 3.63 | ± | 1.01 | 6.57 | ± | 3.22 |
| O-Vanillin | - | ± | - | - | ± | - | - | ± | - | - | ± | - |
| p-Coumaric Acid | 1.02 | ± | 0.21 | 0.95 | ± | 0.10 | 1.02 | ± | 0.40 | 0.59 | ± | 0.00 |
| Salicylic Acid | - | ± | - | - | ± | - | - | ± | - | 0.22 | ± | 0.00 |
| Sebacic Acid | - | ± | - | - | ± | - | - | ± | - | - | ± | - |
| Vanillin | 0.24 | ± | 0.00 | 0.67 | ± | 0.39 | 0.08 | ± | 0.08 | - | ± | - |
| Vanillylamide | 3.20 | ± | 0.27 | 3.70 | ± | 0.11 | - | ± | - | - | ± | - |

The symbol “-“ indicates that the compound was not detected in that particular sample. ‘Avg’ stands for average; ‘Std Dev’ for standard deviation of the population for n=3.

**Table 9A. Normalized saponin abundance in water extracts of switchgrass extracted before AFEX treatment for paired control and drought-year samples:** Saponin abundance in water extracts for a specific molecular weight with respect to internal standard telmisartan used in non-targeted LC-MS analysis. The number in the first column indicates the ‘elution time (mass of the molecule)’. m/z indicates the mass to charge ratio and ‘n’ indicates the neutral mass of the identified compound in Da. ‘Avg’ stands for average; ‘Std Dev’ for standard deviation of the population; ‘wrt’ for with respect to; ‘MW’ for molecular weight

| Normalised saponin abundance wrt internal standard in water extracts | | | | | | | | | | |
| --- | --- | --- | --- | --- | --- | --- | --- | --- | --- | --- |
| Compound elution time (MW) | Annotation | Untreated | | | | | | | |  |
|  |  | 2010 (Control) | | | | 2012 (Drought) | | | |  |
|  |  | Rep 1 | Rep 2 | Rep 3 | Avg | Rep 1 | Rep 2 | Rep 3 | Avg |  |
| 10.93 (868.4803n) | Saponin 868 (C_45_H_72_O_16_) | 1186 | 1493 | 305 | 995 | 4787 | 2927 | 205 | 2640 |  |
| 9.67 (1032.5694m/z) | Saponin 1014 (C_51_H_82_O_20_) isomer 1 | 51 | 58 | 16 | 42 | 231 | 218 | 13 | 154 |  |
| 10.21 (1014.5364n) | Saponin 1014 (C_51_H_82_O_20_) isomer 2 | 4105 | 4389 | 1232 | 3242 | 9024 | 8422 | 988 | 6145 |  |
| 8.72 (1048.5655m/z) | Saponin 1030 (C_51_H_82_O_21_) | 273 | 246 | 230 | 250 | 178 | 352 | 128 | 219 |  |
| 6.09 (1176.5903n) | Saponin 1176 (C_57_H_92_O_25_) | 36452 | 36744 | 16455 | 29883 | 185948 | 207659 | 63332 | 152313 |  |
| 7.05 (1193.6148n) | Saponin 1194 (C_57_H_94_O_26_) | 672 | 525 | 561 | 586 | 7935 | 9925 | 254 | 6038 |  |
| 6.11 (1212.6343m/z) | Unknown saponin | 19828 | 19109 | 17641 | 18859 | 100374 | 116280 | 75302 | 97319 |  |
| 6.11 (1195.0993n) | Unknown saponin | 634 | 709 | 408 | 584 | 12135 | 14196 | 4359 | 10230 |  |
| 9.49 (1356.6750m/z) | Saponin 1338 (C_63_H_102_O_30_) | 489 | 517 | 185 | 397 | 4100 | 4612 | 542 | 3084 |  |

**Table 9B. Normalized saponin abundance in water extracts of switchgrass extracted after AFEX treatment for paired control and drought-year samples:** Saponin abundance in water extracts for a specific molecular weight with respect to internal standard telmisartan used in non-targeted LC-MS analysis. The number in the first column indicates the ‘elution time (mass of the molecule)’. m/z indicates the mass to charge ratio and ‘n’ indicates the neutral mass of the identified compound in Da. ‘Avg’ stands for average; ‘Std Dev’ for standard deviation of the population; ‘wrt’ for with respect to; ‘MW’ for molecular weight

| Normalised saponin abundance wrt internal standard in water extracts | | | | | | | | | | |
| --- | --- | --- | --- | --- | --- | --- | --- | --- | --- | --- |
| Compound elution time (MW) | Annotation | AFEX-Pretreated | | | | | | | |  |
|  |  | 2010 (Control) | | | | 2012 (Drought) | | | |  |
|  |  | Rep 1 | Rep 2 | Rep 3 | Avg | Rep 1 | Rep 2 | Rep 3 | Avg |  |
| 10.93 (868.4803n) | Saponin 868 (C_45_H_72_O_16_) | 7543 | 2140 | 1436 | 3706 | 6429 | 571 | 1269 | 2756 |  |
| 9.67 (1032.5694m/z) | Saponin 1014 (C_51_H_82_O_20_) isomer 1 | 302 | 75 | 61 | 146 | 742 | 27 | 67 | 279 |  |
| 10.21 (1014.5364n) | Saponin 1014 (C_51_H_82_O_20_) isomer 2 | 21864 | 12198 | 8875 | 14312 | 27821 | 6799 | 14230 | 16283 |  |
| 8.72 (1048.5655m/z) | Saponin 1030 (C_51_H_82_O_21_) | 2284 | 1555 | 1108 | 1649 | 2283 | 233 | 622 | 1046 |  |
| 6.09 (1176.5903n) | Saponin 1176 (C_57_H_92_O_25_) | 106739 | 70982 | 56720 | 78147 | 256321 | 59654 | 112863 | 142946 |  |
| 7.05 (1193.6148n) | Saponin 1194 (C_57_H_94_O_26_) | 2761 | 3979 | 2681 | 3140 | 12866 | 827 | 1980 | 5224 |  |
| 6.11 (1212.6343m/z) | Unknown saponin | 56886 | 83094 | 65253 | 68411 | 143291 | 68368 | 139975 | 117211 |  |
| 6.11 (1195.0993n) | Unknown saponin | 3430 | 3845 | 2838 | 3371 | 15698 | 3579 | 9905 | 9727 |  |
| 9.49 (1356.6750m/z) | Saponin 1338 (C_63_H_102_O_30_) | 2487 | 1319 | 783 | 1530 | 7003 | 848 | 2072 | 3308 |  |

**Table 10A. Normalized saponin abundance in ethanol extracts of switchgrass extracted before AFEX treatment for paired control and drought-year samples:** Saponin abundance in ethanol extracts for a specific molecular weight with respect to internal standard telmisartan used in non-targeted LC-MS analysis. The number in the first column indicates the ‘elution time (mass of the molecule)’. m/z indicates the mass to charge ratio and ‘n’ indicates the neutral mass of the identified compound in Da. ‘Avg’ stands for average; ‘Std Dev’ for standard deviation of the population; ‘wrt’ for with respect to; ‘MW’ for molecular weight

| Normalised saponin abundance wrt internal standard in ethanol extracts | | | | | | | | | |
| --- | --- | --- | --- | --- | --- | --- | --- | --- | --- |
| Compound elution time (MW) | Annotation | Untreated | | | | | | | |
|  |  | 2010 (Control) | | | | 2012 (Drought) | | | |
|  |  | Rep 1 | Rep 2 | Rep 3 | Avg | Rep 1 | Rep 2 | Rep 3 | Avg |
| 10.93 (868.4803n) | Saponin 868 (C_45_H_72_O_16_) | 12106 | 11653 | 2089 | 8616 | 11698 | 13395 | 2264 | 9119 |
| 9.67 (1032.5694m/z) | Saponin 1014 (C_51_H_82_O_20_) isomer 1 | 37 | 33 | 44 | 38 | 27 | 35 | 47 | 37 |
| 10.21 (1014.5364n) | Saponin 1014 (C_51_H_82_O_20_) isomer 2 | 21322 | 18024 | 10857 | 16734 | 25242 | 16773 | 12941 | 18319 |
| 8.72 (1048.5655m/z) | Saponin 1030 (C_51_H_82_O_21_) | 2701 | 2505 | 523 | 1910 | 1534 | 1617 | 306 | 1152 |
| 6.09 (1176.5903n) | Saponin 1176 (C_57_H_92_O_25_) | 22048 | 21296 | 2679 | 15341 | 145103 | 158248 | 27037 | 110129 |
| 7.05 (1193.6148n) | Saponin 1194 (C_57_H_94_O_26_) | 13289 | 12765 | 2792 | 9615 | 5725 | 5449 | 1246 | 4140 |
| 6.11 (1212.6343m/z) | Unknown saponin | 11934 | 12124 | 2592 | 8884 | 84526 | 84988 | 29988 | 66500 |
| 6.11 (1195.0993n) | Unknown saponin | 323 | 391 | 33 | 249 | 8090 | 7921 | 1177 | 5729 |
| 9.49 (1356.6750m/z) | Saponin 1338 (C_63_H_102_O_30_) | 2224 | 2033 | 388 | 1548 | 4206 | 4158 | 769 | 3044 |

**Table 10B. Normalized saponin abundance in ethanol extracts of switchgrass extracted after AFEX treatment for paired control and drought-year samples:** Saponin abundance in ethanol extracts for a specific molecular weight with respect to internal standard telmisartan used in non-targeted LC-MS analysis. The number in the first column indicates the ‘elution time (mass of the molecule)’. m/z indicates the mass to charge ratio and ‘n’ indicates the neutral mass of the identified compound in Da. ‘Avg’ stands for average; ‘Std Dev’ for standard deviation of the population; ‘wrt’ for with respect to; ‘MW’ for molecular weight

| Normalised saponin abundance wrt internal standard in ethanol extracts | | | | | | | | | | |
| --- | --- | --- | --- | --- | --- | --- | --- | --- | --- | --- |
| Compound elution time (MW) | Annotation | AFEX-Pretreated | | | | | | | |  |
|  |  | 2010 (Control) | | | | 2012 (Drought) | | | |  |
|  |  | Rep 1 | Rep 2 | Rep 3 | Avg | Rep 1 | Rep 2 | Rep 3 | Avg |  |
| 10.93 (868.4803n) | Saponin 868 (C_45_H_72_O_16_) | 22778 | 4230 | 3696 | 10235 | 14534 | 3432 | 3269 | 7079 |  |
| 9.67 (1032.5694m/z) | Saponin 1014 (C_51_H_82_O_20_) isomer 1 | 417 | 92 | 97 | 202 | 440 | 108 | 95 | 214 |  |
| 10.21 (1014.5364n) | Saponin 1014 (C_51_H_82_O_20_) isomer 2 | 59494 | 21738 | 18245 | 33159 | 49454 | 26126 | 24880 | 33487 |  |
| 8.72 (1048.5655m/z) | Saponin 1030 (C_51_H_82_O_21_) | 4091 | 830 | 692 | 1871 | 2296 | 552 | 532 | 1127 |  |
| 6.09 (1176.5903n) | Saponin 1176 (C_57_H_92_O_25_) | 61354 | 6858 | 5235 | 24482 | 156556 | 29009 | 26356 | 70640 |  |
| 7.05 (1193.6148n) | Saponin 1194 (C_57_H_94_O_26_) | 23702 | 4977 | 4292 | 10990 | 13913 | 3149 | 2999 | 6687 |  |
| 6.11 (1212.6343m/z) | Unknown saponin | 33586 | 7631 | 5464 | 15560 | 81892 | 31912 | 29314 | 47706 |  |
| 6.11 (1195.0993n) | Unknown saponin | 1542 | 117 | 82 | 581 | 8125 | 1177 | 1049 | 3450 |  |
| 9.49 (1356.6750m/z) | Saponin 1338 (C_63_H_102_O_30_) | 6092 | 1022 | 791 | 2635 | 11456 | 3001 | 2588 | 5682 |  |

**Table 11A. Normalized saponin abundance in ethyl acetate extracts of switchgrass extracted before AFEX treatment for paired control and drought-year samples:** Saponin abundance in ethyl acetate extracts for a specific molecular weight with respect to internal standard telmisartan used in non-targeted LC-MS analysis. The number in the first column indicates the ‘elution time (mass of the molecule)’. m/z indicates the mass to charge ratio and ‘n’ indicates the neutral mass of the identified compound in Da. ‘Avg’ stands for average; ‘Std Dev’ for standard deviation of the population; ‘wrt’ for with respect to; ‘MW’ for molecular weight

| Normalised saponin abundance wrt internal standard in ethyl acetate extracts | | | | | | | | | | |
| --- | --- | --- | --- | --- | --- | --- | --- | --- | --- | --- |
| Compound elution time (MW) | Annotation | Untreated | | | | | | | |  |
|  |  | 2010 (Control) | | | | 2012 (Drought) | | | |  |
|  |  | Rep 1 | Rep 2 | Rep 3 | Avg | Rep 1 | Rep 2 | Rep 3 | Avg |  |
| 10.93 (868.4803n) | Saponin 868 (C_45_H_72_O_16_) | 7122 | 5781 | 1509 | 4804 | 4271 | 4227 | 4294 | 4264 |  |
| 9.67 (1032.5694m/z) | Saponin 1014 (C_51_H_82_O_20_) isomer 1 | 54 | 34 | 0 | 29 | 41 | 47 | 44 | 44 |  |
| 10.21 (1014.5364n) | Saponin 1014 (C_51_H_82_O_20_) isomer 2 | 18509 | 19290 | 7567 | 15122 | 16847 | 15930 | 14473 | 15750 |  |
| 8.72 (1048.5655m/z) | Saponin 1030 (C_51_H_82_O_21_) | 1198 | 1183 | 338 | 907 | 384 | 347 | 556 | 429 |  |
| 6.09 (1176.5903n) | Saponin 1176 (C_57_H_92_O_25_) | 322 | 336 | 61 | 240 | 3818 | 4164 | 2585 | 3522 |  |
| 7.05 (1193.6148n) | Saponin 1194 (C_57_H_94_O_26_) | 1523 | 1598 | 429 | 1183 | 643 | 647 | 725 | 672 |  |
| 6.11 (1212.6343m/z) | Unknown saponin | 150 | 180 | 35 | 122 | 2203 | 2348 | 2902 | 2484 |  |
| 6.11 (1195.0993n) | Unknown saponin | 0 | 0 | 0 | 0 | 26 | 36 | 43 | 35 |  |
| 9.49 (1356.6750m/z) | Saponin 1338 (C_63_H_102_O_30_) | 6 | 6 | 0 | 4 | 34 | 24 | 89 | 49 |  |

**Table 11B. Normalized saponin abundance in ethyl acetate extracts of switchgrass extracted after AFEX treatment for paired control and drought-year samples:** Saponin abundance in ethyl acetate extracts for a specific molecular weight with respect to internal standard telmisartan used in non-targeted LC-MS analysis. The number in the first column indicates the ‘elution time (mass of the molecule)’. m/z indicates the mass to charge ratio and ‘n’ indicates the neutral mass of the identified compound in Da. ‘Avg’ stands for average; ‘Std Dev’ for standard deviation of the population; ‘wrt’ for with respect to; ‘MW’ for molecular weight

| Normalised saponin abundance wrt internal standard in ethyl acetate extracts | | | | | | | | | | |
| --- | --- | --- | --- | --- | --- | --- | --- | --- | --- | --- |
| Compound elution time (MW) | Annotation | AFEX-Pretreated | | | | | | | |  |
|  |  | 2010 (Control) | | | | 2012 (Drought) | | | |  |
|  |  | Rep 1 | Rep 2 | Rep 3 | Avg | Rep 1 | Rep 2 | Rep 3 | Avg |  |
| 10.93 (868.4803n) | Saponin 868 (C_45_H_72_O_16_) | 4890 | 2205 | 2530 | 3209 | 70149 | 1874 | 1772 | 24598 |  |
| 9.67 (1032.5694m/z) | Saponin 1014 (C_51_H_82_O_20_) isomer 1 | 51 | 29 | 43 | 41 | 224 | 20 | 21 | 88 |  |
| 10.21 (1014.5364n) | Saponin 1014 (C_51_H_82_O_20_) isomer 2 | 17423 | 9742 | 11714 | 12960 | 17004 | 11585 | 10938 | 13175 |  |
| 8.72 (1048.5655m/z) | Saponin 1030 (C_51_H_82_O_21_) | 494 | 273 | 318 | 361 | 849 | 134 | 128 | 371 |  |
| 6.09 (1176.5903n) | Saponin 1176 (C_57_H_92_O_25_) | 454 | 0 | 0 | 151 | 468 | 27 | 28 | 174 |  |
| 7.05 (1193.6148n) | Saponin 1194 (C_57_H_94_O_26_) | 495 | 218 | 238 | 317 | 1764 | 92 | 86 | 647 |  |
| 6.11 (1212.6343m/z) | Unknown saponin | 225 | 0 | 0 | 75 | 220 | 18 | 26 | 88 |  |
| 6.11 (1195.0993n) | Unknown saponin | 0 | 0 | 0 | 0 | 0 | 0 | 0 | 0 |  |
| 9.49 (1356.6750m/z) | Saponin 1338 (C_63_H_102_O_30_) | 0 | 0 | 0 | 0 | 143 | 0 | 0 | 48 |  |

**References:**

- Ong RG, Higbee A, Bottoms S, Dickinson Q, Xie D, Smith SA, Serate J, Pohlmann E, Jones AD, Coon JJ, Sato TK, Sanford GR, Eilert D, Oates LG, Piotrowski JS, Bates DM, Cavalier D, and Zhang Y. Inhibition of microbial biofuel production in drought-stressed switchgrass hydrolysate*.* *Biotechnol. Biofuels* (2016) **9**(1):237. <http://dx.doi.org/10.1186/s13068-016-0657-0>
